# An In Vitro Study of Saffron Carotenoids: The Effect of Crocin Extracts and Dimethylcrocetin on Cancer Cell Lines

**DOI:** 10.3390/antiox11061074

**Published:** 2022-05-28

**Authors:** Kyriaki Hatziagapiou, Olti Nikola, Sofia Marka, Eleni Koniari, Eleni Kakouri, Maria-Eleftheria Zografaki, Sophie S. Mavrikou, Charalabos Kanakis, Emmanouil Flemetakis, George P. Chrousos, Spyridon Kintzios, George I. Lambrou, Christina Kanaka-Gantenbein, Petros A. Tarantilis

**Affiliations:** 1 Choremeio Research Laboratory, First Department of Pediatrics, National and Kapodistrian University of Athens, Thivon & Levadeias 8, 11527 Athens, Greece; onikola@med.uoa.gr (O.N.); ckanaka@med.uoa.gr (C.K.-G.); 2 Physiotherapy Department, Faculty of Health and Care Sciences, State University of West Attica, Agiou Spiridonos 28, Egaleo, 12243 Athens, Greece; 3 Laboratory of Cell Technology, Department of Biotechnology, Agricultural University of Athens, EU-CONEXUS European University, 11855 Athens, Greece; smarka@aua.gr (S.M.); sophie_mav@aua.gr (S.S.M.); skin@aua.gr (S.K.); 4 University Research Institute of Maternal and Child Health & Precision Medicine, UNESCO Chair on Adolescent Health Care, National and Kapodistrian University of Athens, Thivon & Levadeias 8, 11527 Athens, Greece; hkoniari@med.uoa.gr (E.K.); chrousge@med.uoa.gr (G.P.C.); 5 Laboratory of Chemistry, Department of Food Science & Human Nutrition, School of Food Biotechnology and Development, Agricultural University of Athens, EU-CONEXUS European University, 11855 Athens, Greece; elenikakouri@aua.gr (E.K.); chkanakis@aua.gr (C.K.); ptara@aua.gr (P.A.T.); 6 Laboratory of Molecular Biology, Department of Biotechnology, Agricultural University of Athens, EU-CONEXUS European University, 11855 Athens, Greece; mzografaki@aua.gr (M.-E.Z.); mflem@aua.gr (E.F.)

**Keywords:** crocins (CRCs), dimethylocrocetin (DMCRT), saffron, glioblastoma (GBM), rhabdomyosarcoma, cytotoxicity

## Abstract

*Crocus sativus* L. has various pharmacological properties, known for over 3600 years. These properties are attributed mainly to biologically active substances, which belong to the terpenoid group and include crocins, picrocrocin and safranal. The aim of the current work was to examine the effects of crocins (CRCs) and their methyl ester derivate dimethylcrocetin (DMCRT) on glioblastoma and rhabdomyosarcoma cell lines, in terms of cytotoxicity and gene expression, implicated in proapoptotic and cell survival pathways. Cell cytotoxicity was assessed with Alamar Blue fluorescence assay after treatment with saffron carotenoids for 24, 48 and 72 h and concentrations ranging from 22.85 to 0.18 mg/mL for CRCs and 11.43 to 0.09 mg/mL for DMCRT. In addition, *BAX*, *BID*, *BCL2*, *MYCN*, *SOD1*, and *GSTM1* gene expression was studied by qRT-PCR analysis. Both compounds demonstrated cytotoxic effects against glioblastoma and rhabdomyosarcoma cell lines, in a dose- and time-dependent manner. They induced apoptosis, via *BAX and BID* upregulation, *MYCN* and *BCL-2*, *SOD1*, *GSTM1* downregulation. The current research denotes the possible anticancer properties of saffron carotenoids, which are considered safe phytochemicals, already tested in clinical trials for their health promoting properties.

## 1. Introduction

The World Health Organization has declared that cancer currently constitutes a major public health challenge worldwide, accounting for one in six deaths, that is nearly 10 million deaths globally, in 2020. The most worrying fact is that the number of new cases and deaths are estimated to rise significantly due to the global trend for longevity, epidemiological and demographic transitions and the adoption of cancer-associated choices, including smoking, sedentary lifestyle and ‘‘westernized’’ diets; hence, the global burden of all cancers is predicted to reach 27.5–37 million new cases/year by 2040 [1,2,3,4]. Surgery, radiotherapy and chemotherapy represent the mainstay of medical oncology therapeutics, however the disadvantage with the last two treatment modalities is the lack of selectivity and the insult of vital cellular targets shared by both cancer and normal cells, e.g., cell cycle and metabolism, DNA synthesis and repair and protein synthesis [5]. Thus, cancer survivors usually suffer from serious and sometimes, life-threatening short- or long-term complications, e.g., myelosuppression, secondary tumors, gastrointestinal toxicity, alopecia, cardiotoxicity, lung fibrosis and cognitive impairment. It is nowadays evident that conventional chemotherapy and radiation therapy have reached a plateau on efficacy as sole and/or primary treatment choices, in terms of cancer remission, survival rates and tolerance in side effects.

Medicinal plants, their secondary metabolites and synthesized chemical compounds still provide some of the most original and promising approaches for discovering new anticancer therapeutic agents. It is nevertheless noteworthy that 60% of commercially available antitumor therapies derive from natural sources; e.g., Vinca alkaloids (vinorelbine, vindesine, vincristine and vinblastine) were isolated from *Catharanthus roseus* (Madagascar Periwinkle), the taxane paclitaxel from the bark and leaf of *Taxus baccata* and *Taxus canadensis* and its semi-synthetic derivative docetaxel from *T. baccata*, Campothecin (topoisomerase I inhibitor) from *Camptotheca acuminata*, etoposide and teniposide from *Podophyllum peltatum* (May Apple) [6,7,8]. The growing burden of cancer worldwide, along with several limitations in conventional therapies, including high cost and severe toxicities, has led the research towards natural compounds, as they provide a huge variety, whereas some of them are endowed with strong biological activities.

There is a growing body of compelling evidence that *Crocus sativus* L. could be considered a medicinal plant, probably possessing anticancer, antioxidant, anti-inflammatory, hepatoprotective, antiatherosclerotic, hypolipidemic, hypotensive, antidiabetic, anticonvulsant, antidepressant, anxiolytic and hypnotic effects, improver of cognitive performance and memory impairment [9,10,11,12]. The red stigmas of *Crocus sativus* L., commonly named as saffron have three major characteristic components: (i) crocins (CRCs) (8′-diapocarotene-8,8′-dioic acid), which are mainly responsible for the red pigmentation of stigmas; (ii) picrocrocin (C_16_H_26_O_7_), a monoterpene glycoside of the aglycone 4-hydroxy-2,6,6-trimethyl-1-carboxaldehyde-1-cyclohexene (HTCC), responsible for its distinctive bitter flavor; and (iii) safranal (2,6,6-trimethylcyclohexane-1,3-dien-1-carboxaldehyde) (C_10_H_14_O), an aromatic monoterpene aldehyde and the main component of the essential oil, providing the characteristic odor to saffron. Other constituents include carotenoids (alpha-, beta-, gamma-carotene, lycopene, zeaxanthin and xanthone-carotenoid glycosidic conjugate), phenolic and flavonoid compounds [13,14,15,16,17,18,19,20].

CRCs are water soluble carotenoids and the prevailing constituent of *Crocus sativus* L. stigmas, primarily responsible for the pharmacologic activity of saffron [21]. They are either mono- or di-glycosyl esters of a dicarboxylic acid crocetin (CRT; 2,6,11,15-tetramethylhexadeca-2,4,6,8,10,12,14-heptaenedioic acid; C_20_H_24_O_4_), in which d-glucose and/or d-gentiobiose occur as carbohydrate residues. The methyl ester derivate dimethylcrocetin (DMCRT) is similar to CRT. Their difference is attributed to the presence of a methoxy group at positions C-1 and C-16 of the carbon chain instead of the carboxyl group found in CRT. DMCRT is soluble in many organic solvents acceptable in cell experiments [13,14,18,22].

Constituents and metabolites of saffron are relatively safe as demonstrated in several studies on animal models, in terms of acute, subacute, sub chronic and chronic exposure. Saffron and its metabolites did not cause any significant hematological or biochemical toxic effects, nor were associated with histotoxicity in heart, liver, spleen and kidney, or genotoxic and mutagenic effects [23,24,25,26,27,28,29,30,31,32]. CRCs in preclinical mice models were well tolerated in doses up to 3 g/kg, in intraperitoneal or oral administration for acute exposure and 400 mg/kg in intraperitoneal administration for subacute exposure (3–4 weeks) [26,33,34,35,36,37,38,39,40,41,42]. 

Saffron and its constituents have been studied for their potential anticancer and antioxidant properties and their cytotoxic effect on several cancer cell lines [43,44,45,46,47,48,49]. However, there is not any evidence on the effects of CRCs and DMCRT on glioblastoma (GBM) and rhabdomyosarcoma cell lines. Therefore, the aim of the current work is to examine, using biological assays, the effects of CRCs and DMCRT on GBM and rhabdomyosarcoma tumor cell lines, in terms of cytotoxicity and alterations on the expression of key signaling molecules, implicated in proapoptotic pathways and of cellular antioxidants.

## 2. Materials and Methods

### 2.1. Extraction of Crocins and Dimethylcrocetin

CRCs were extracted from the stigmas of *Crocus sativus* L. (5 g of dried stigmas) as previously described [50]. The chemical profile of CRCs has been elucidated in previous studies [16,50,51,52,53,54]. At this study, the analysis of the extract was re-evaluated using the same technique and under the same operation parameters as reported in the study of Kakouri et al. (2020) [50]. In particular, samples were analyzed using an LC/Q-TOF/HRMS technique (Agilent Series 1260-Agilent Technologies, Boeblingen, Germany). Identification of the compounds detected was based on literature and UV data [16,50,51,52,53,54]. Identification was further ascertained by the fact that a Q-TOF analyzer provides the chemical formula of each detected compound based on accurate mass measurement, which in our study, was adjusted to less than 5 ppm. Nomenclature of the identified compounds was based on the proposal of Carmona et al., i.e., the first part describes the cis/trans form of the aglycon part, then follows the total number of sugar moieties (glycose monomers) and finally is indicated the type of sugar in each part of crocin structure [51].

In the case of DMCRT, five grams of powdered dried stigmas were first extracted with 50 mL of petroleum ether and after that with diethyl ether. Extraction took place 5 times for each solvent used in an ultrasound water bath, at 25 °C and at 35 kHz ultrasound frequency for 10 min each (total time 50 min, i.e., [5 times for each solvent] × 10 min). The received powder was dried under nitrogen steam and extracted with 200 mL of methanol (HPLC grade). The extract was left on a magnetic stirrer for 120 min, protected from light. Extraction with methanol was repeated under the same conditions twice. The extract (400 mL) was kept at 4 °C and a new extraction followed, where the extract remained under stirring for 24 h. Extracts were then combined (600 mL final volume) and alkaline hydrolysis followed. An aqueous KOH solution (2N) was gradually added to the final extract under continuous stirring, until solid particles were formed, indicating the presence of DMCRT. The received extract was centrifuged (SIGMA 3K) at 8000 rpm (7155× *g*) for 15 min and the solid residue was collected. Chloroform was then added in order to receive a more purified extract and evaporation of the organic solvent took place. The purity of the final material was tested with the diffuse reflectance infrared Fourier transform (DRIFT), using a Nicolet 6700 FTIR spectrometer operating at the region of 4000–400 cm^–1^ and equipped with a deuterated triglycine sulphate detector (DTGs). Spectra were recorded with a total of 100 scans at a resolution of 4 cm^−1^, using the Diffuse Reflectance Infrared Fourier Transform Spectroscopy (DRIFTs) technique. Baseline correction and smoothing of the generated spectra took place before analysis of the data, which was performed using the OMNIC 3.1 software. Characteristic peaks at 1696 cm^−1^, 1225 and 1106 cm^−1^ confirmed the presence of DMCRT [55]. The final powdered material was kept at 4 °C until further use.

### 2.2. Cell Culture and Reagents

A172 glioblastoma (ECACC 88062428) and TE-671 (ECACC 89071904) rhabdomyosarcoma cell lines were purchased from the European Collection of Cell Cultures (ECACC, London, UK). Cell lines were maintained as exponentially growing monolayer cultures in DMEM, supplemented with 1% l-Glutamine-penicillin/streptomycin (Invitrogen-Gibco Inc., Carlsbad, CA, USA) and 15% fetal bovine serum (FBS) (Invitrogen-Gibco Inc., Carlsbad, CA, USA) in the Steri-Cycle CO_2_ incubator (Thermo Scientific, Waltham, MA, USA) at 37 °C, 95% humidity and 5% CO_2_. Experiments were conducted in the laminar flow hood (ESI-FLUFRANCE, Wissous, France) to reduce the risk of cell contamination. The culture medium was renewed, depending on the needs of cell growth, usually every three days to remove toxic metabolic derivatives, which could interfere with normal cell growth and proliferation.

Cells were allowed to grow at ~80% confluence and harvested using 0.1% trypsin and centrifugation at 2400 rpm (708× *g*) for 10 min. Supernatant was discarded and pellet was kept for further processing. Cell population was determined with the use of a NIHON KOHDEN CellTaq-a (Nihon Kohden EUROPE GmbH, Rosbach, Germany) hematology analyzer.

### 2.3. Experimental Setup

Experiments were performed in 96-well plates (CellStar^®^, Sigma-Aldrich Chemie GmbH, Taufkirchen, DE, USA). Experimental setup included a column of cell culture medium only, a column of cell culture medium and the respective staining chemical, a column of cultured cells only (no drug, nor staining agent) and a column of cultured cells and the respective staining chemical, whereas the remaining wells were used for the testing of several concentrations of the testing agents (CRCs, DMCRT). Wells containing cell culture medium only and cells with no staining agent or drug (untreated cells) were used as blank, whereas wells with cultured cells with staining agent were used as positive controls.

### 2.4. Assessment of Cell Viability

CRCs were diluted to nuclease and protease free water and DMCRT to 5% DMSO. In order to prepare different concentrations of each testing agent, serial dilutions were performed. Specifically, for DMCRT the successive dilutions led to a final concentration of DMSO in the testing wells lower than 1%. Cell viability was assessed after incubation with the testing agents via resazurin reduction experiments, using an Alamar Blue viability assay (Invitrogen-Gibco Inc., Carlsbad, CA, USA), as previously described [56,57]. In brief, cells were cultured in 96-well plates (Cellstar, Greiner GmbH, Frickenhausen, Germany) at a density of 2 × 10^4^ cells (*C* = 10^5^ cells/lt) per well in their respective medium, following 24-h incubation at 37 °C, until reaching confluency. Then, saffron compounds were added separately to the wells by serial dilution to final concentrations of 22.85, 11.429, 5.714, 2.857, 1.429, 0.714, 0.357 and 0.179 mg/mL for CRCs and 11.429, 5.714, 2.857, 1.429, 0.714, 0.357, 0.179 and 0.090 mg/mL for DMCRT. Higher doses than 22.85 mg/mL and 11.429 mg/mL for CRCs and DMCRT respectively were not applied, as at higher doses the compounds would not dilute and precipitated. Cells were incubated with the aforementioned phytochemicals for 24, 48 and 72 h.

To quantify the number of viable cells at each time point (24, 48 and 72 h) of exposure, Alamar Blue was added to wells, to a final concentration of 10% and cells were incubated for 6 h. The reduced form of Alamar Blue was determined by fluorescence measurements at excitation wavelength 550 nm and emission of 590 nm. Measurements were carried out using an automated-microtiter plate reader Victor^3^ (Perkin Elmer Inc., Waltham, MA, USA). Signal intensities were normalized by subtracting the blank signal intensity from the signal intensity of the experimental wells. Each drug testing was performed in triplicates and performed at least two independent times.

### 2.5. RNA Extraction and cDNA Synthesis

A172 glioblastoma and TE671 rhabdomyosarcoma cells were cultured in 25 cm^2^ flasks and incubated for 24, 48 and 72 h with CRCs and DMCRT. CRCs were diluted in cell culture media, while DMCRT was dissolved in DMSO, with its final concentration in flasks not exceeding 1%. Each reagent was added to the flasks in a final concentration of 1 mg/mL and left for incubation at 37 °C for 48 and 72 h. RNA isolation/purification was carried out at each time point. Total RNA isolation/ purification was performed using QIAGEN RNeasy Mini Kit (Qiagen GmbH, Hilden, Germany) according to the manufacturer’s instructions. cDNA synthesis was performed using 500 ng of total RNA, using a QuantiTect Reverse Transcription Kit (Qiagen GmbH, Hilden, Germany) protocol in a total volume of 20 μL. The reaction mixture was incubated at 42 °C for 50 min, followed by heat inactivation at 70 °C for 15 min. The complementary DNA (cDNA) was stored at −20 °C until further use.

### 2.6. Reverse Transcription Real-Time PCR (qRT-PCR)

The StepOnePlus Real-Time PCR System (Applied Biosystems, Foster City, CA, USA) was used for conducting the RT-qPCR analysis while SYBR™ Select Master Mix (Applied Biosystems, Foster City, USA) was utilized for target genes expression. Primers pairs were designed using PrimerExpress 2.0 software (Thermo Fisher Scientific, Waltham, MA, USA) and their sequences are shown in Table 1. In order to confirm the reaction specificity, dissociation curve analysis was demonstrated. The expression levels of Beta-Actin (ACTB) and glyceraldehyde-3-phosphate dehydrogenase (GAPDH) were used as reference genes. The reaction started at 95 °C for a 15 min time period, followed by 40 cycles of 95 °C for 15 s and 60 °C for 1 min. The calculation of the relative transcript levels of the gene of interest was made by the comparative threshold cycle (CT) method [(1 + E)^−ΔΔCT^] [58]. LinRegPCR software was used for the calculation of PCR efficiency for each amplicon [59]. All the results were expressed as mean ± SEM. One-way analysis of variance (ANOVA) following by Tukey’s multiple comparisons test was used to detect statistical differences. The level of significant difference was set at *p*-value < 0.05. The SigmaPlot software 12.0 (Systat Software Inc., San Jose, CA, USA) was used to carry out the statistical analysis.

### 2.7. Data Analysis and Statistics

Multiparameter analyses were performed with GraphPad Prism 8 version 8.0.0 (GraphPad Software for Windows, San Diego, CA, USA, www.graphpad.com; accessed on 21 April 2022). Results in cytotoxicity are expressed as the mean ± SD. of triplicate plates. Each experiment was repeated at least twice. Multiple comparisons two-way ANOVA was used to calculate the significance of the mean differences between groups. *p*-values < 0.05 were considered statistically significant and confidence intervals were at ±95% confidence intervals (±95% CI). Continuous variables are expressed as the mean ± standard deviation (SD), unless otherwise indicated. Nonlinear regressions were performed using the equation:

(1) $$Y=Bottom+\frac{Top-Bottom}{1+10^{X-\log IC_{50}}}.$$

A four parameter logistic model was used to evaluate *IC*_50_, using the equation:

(2) $$Fifty=\frac{Top+Baseline}{2Y}=\frac{Bottom+(Top-Bottom)}{1+10^{(\log IC_{50}-X)\cdot HillSlope+\log\left(\frac{Top-Bottom}{Fifty\_Bottom-1}\right)}}.$$

The percentage of cell viability was calculated as follows:

(3) $$\frac{\mathrm{FL}{\left(nm\right)}_{Cells\_Exposed\_to\_drug}-\mathrm{FL}{\left(nm\right)}_{wells\_containing\_only\_medium}}{\mathrm{FL}{\left(nm\right)}_{Cells\_Not\_Exposed\_to\_drug}-\mathrm{FL}{\left(nm\right)}_{wells\_containing\_only\_medium}}\times 100,$$

where FL is Fluorescence in nm.

Gene Ontology (GO) enrichment was performed using the gprofiler [60] and WebGestalt web tools [61]. 

Finally, we calculated the “velocity” of the cell viability (i.e., the rate by which cell viability changes) by calculating the first derivative of cell viability with respect to time as follows:

(4) $$\frac{dCell\_Viability}{dt}=\frac{Cell\_Viability_{72h}-Cell\_Viability_{24h}}{72h-24h}.$$

## 3. Results

### 3.1. LC/Q-TOF/HRMS Analysis

The compounds detected at the extract were the following: cis/trans crocin-5, cis/trans crocin-4, cis/trans crocin 2, cis/trans crocin-3 and trans crocin-1. Our results are in accordance with previously reported literature data [16]. Trans crocin-4 and trans crocin-3 were detected in abundance, according to the area under the peak, as automatically generated by the Q-TOF analyzer.

### 3.2. The Biological Effects on Glioblastoma Cells (A172)

#### 3.2.1. The Dose-Dependent Effect of CRCs and DMCRT on Glioblastoma Cells (A172)

**Figure 1:**
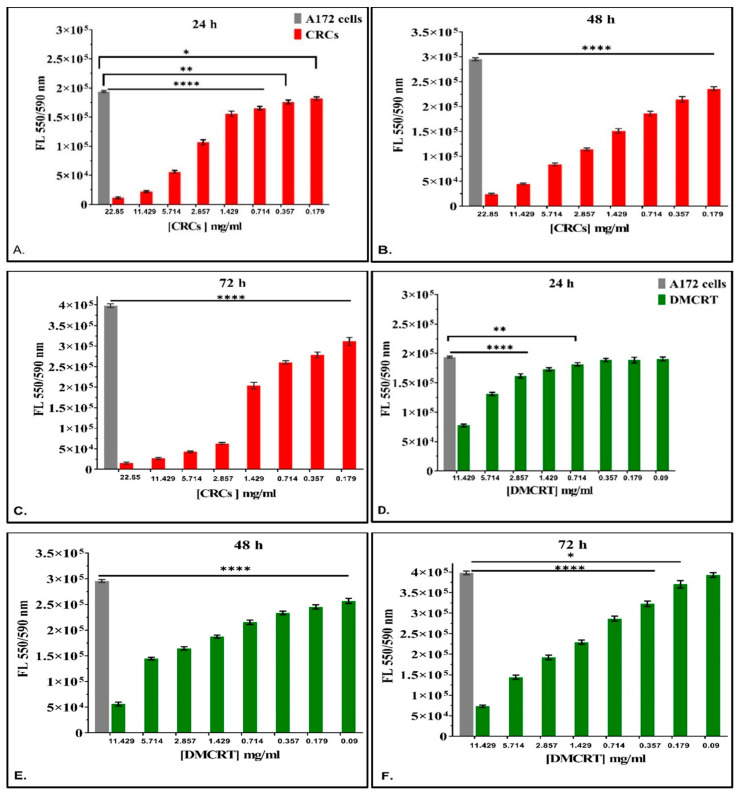
Dose-dependent effect of CRCs and DMCRT on A172 cells. CRCs exhibited a significant dose-dependent effect at all time points and for concentrations ≥0.179 mg/mL tested (*p* < 0.05 for cells exposed to any concentration of CRCs vs untreated A172) (**A**–**C**). Similarly, DMCRT was also effective from concentrations ≥0.714 mg/mL and ≥0.179 mg/mL, at 24 and 72 h respectively and for all concentrations, at 48 h, manifesting significant increasing cell fatal effects with increasing concentrations (**D**–**F**). **Legend**: **CRCs**: Crocins, **DMCRT**: dimethylocrocetin, **FL**: Fluorescence Intensity. Asterisks (*) depict at least *p* < 0.05 significance level between control experiments and CRCs (* *p* < 0.05; ** *p* < 0.01; **** *p* < 0.0001). Each value represents the mean ± S.D. of triplicate experiments.

A172 cells were incubated with CRCs and DMCRT in various concentrations as described in the Section 2. In particular, when analyzing drug effects with respect to concentration for each time interval studied (24, 48 and 72 h) CRCs and DMCRT were indifferent at 0 h of exposure (data not shown), for any concentration, as expected. CRCs (Figure 1A–C) exhibited at all-time points and for concentrations ≥ 0.179 mg/mL a significant dose-dependent cytotoxic effect; the cytotoxicity increased, as the compound’s concentration augmented. DMCRT did not manifest any effect at concentrations ≤ 0.71 mg/mL and 0.09 mg/mL, at 24 and 72 h, respectively, whereas for the rest of concentrations, especially at 48 h there was a clear dose-dependent effect, when compared with the untreated cells (Figure 1D–F).

#### 3.2.2. The Comparative Dose-Dependent Effect of CRCs and DMCRT on Glioblastoma Cells (A172)

**Figure 2:**
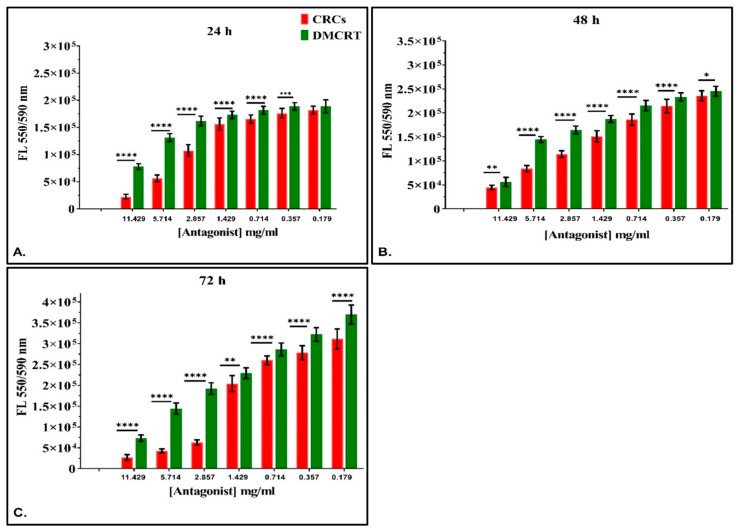
Comparative dose-dependent effect of CRCs and DMCRT on A172 cells. CRCs and DMCRT followed a similar pattern at all time points. CRCs exhibited higher efficacy than DMCRT. This effect was found to be true at all time points that is 24 (**A**), 48 (**B**) and 72 h (**C**) **Legend**: **CRCs**: Crocins, **DMCRT**: dimethylocrocetin, **FL**: Fluorescence Intensity. Asterisks (*) depict at least *p* < 0.05 significance level between control experiments and CRCs (* *p* < 0.05; ** *p* < 0.01; *** *p* < 0.001; **** *p* < 0.0001). Each value represents the mean ± S.D. of triplicate experiments.

Significant differences among CRCs and DMCRT were observed at all time points (24, 48 and 72 h) and for all concentrations; CRCs generally demonstrated better cytotoxic effects as compared to DMCRT. More specifically, CRCs were more potent than DMCRT on A172 cells for concentrations ≥0.36 mg/mL at 24 h (Figure 2A) and for all concentrations at 48-(Figure 2B) and 72-h’ time points (Figure 2C).

#### 3.2.3. The Time-Dependent Effect of CRCs and DMCRT on Glioblastoma Cells (A172)

Further on, the time-dependent efficacy of CRCs and DMCRT was studied, for all concentrations tested. Measurements were performed at 6 h, meaning at the time just after the addition of the drug and the staining agent and every 24 h thereafter i.e., at 24, 48 and 72 h. When examining the drug effect with respect to time for each concentration we observed that at 6 h, as expected, no significant differences were present with respect to any tested bioactive compound (not depicted). 

**Figure 3:**
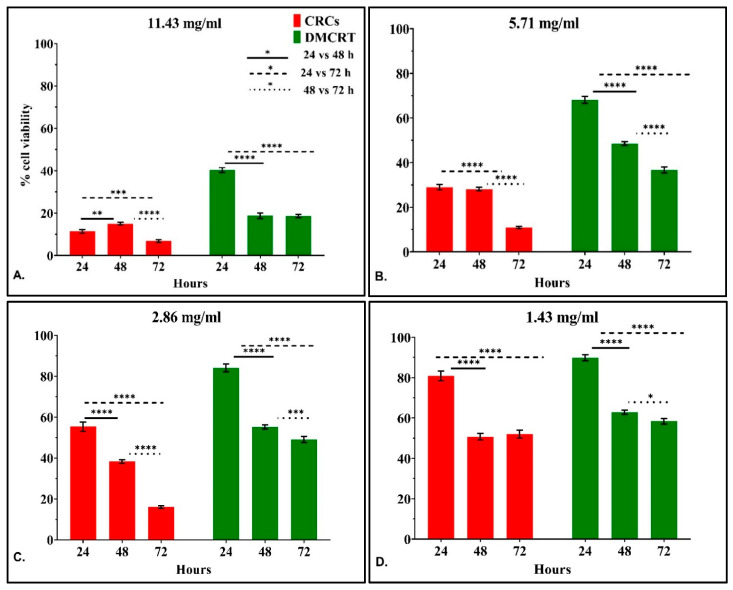

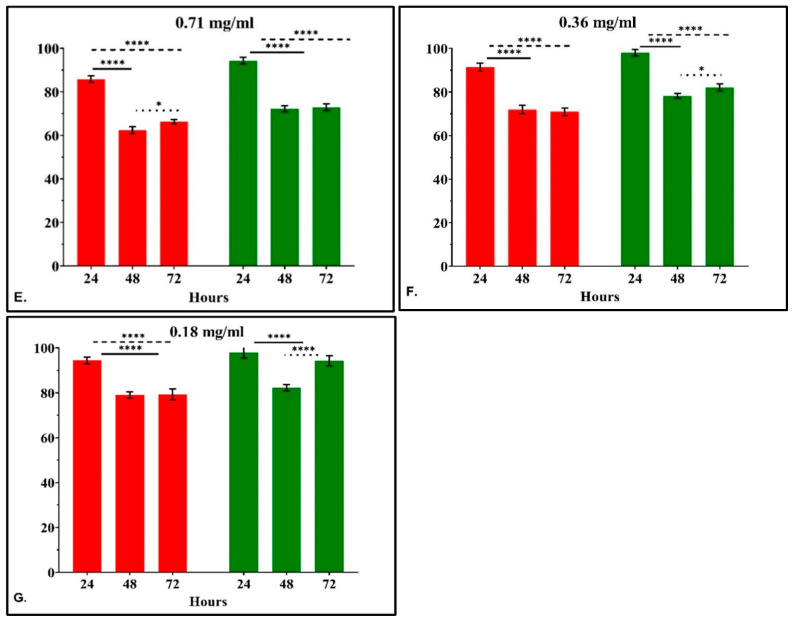
Time-dependent effect of CRCs and DMCRT on A172 cells. Time-dependent effect of CRCs and DMCRT at concentrations 11.429 mg/mL (**A**), 5.174 mg/mL (**B**), 2.857 mg/mL (**C**), 1.429 mg/mL (**D**), 0. 714 mg/mL, (**E**) 0.357 mg/mL (**F**), 0,18 mg/mL (**G**) **Legend**: **CRCs**: Crocins, **DMCRT**: dimethylocrocetin. Asterisks (*) depict at least *p* < 0.05 significance level between control experiments and CRCs (* *p* < 0.05; ** *p* < 0.01; *** *p* < 0.001; **** *p* < 0.0001). Each value represents the mean ± S.D. of triplicate experiments.

CRCs at the high concentrations 2.86–11.43 were more cytotoxic at 72 h, verifying a time-dependent effect (*p* < 0.0001 for 48 vs. 72 h and *p* < 0.0001 for 24 vs. 72 h). For the lowest concentrations 0.18–1.43 mg/mL we observed a significant cytotoxic effect, when comparing 72 vs. 24 h (*p* < 0.0001 for 24 vs. 72 h), whereas for 48 vs. 72 h we did not observe significant differences, indicating that the cytotoxic effect might reach a plateau. When comparing 48 vs. 24 h we observed time-dependent cytotoxicity at concentrations lower than 2.86 mg/mL (Figure 3A–G).

DMCRT manifested a significant time-dependent cytotoxic effect for all concentrations, when comparing 48 vs. 24 h (*p* < 0.0001 for 24 vs. 48 h) and for concentrations ≥ 0.36 mg/mL, when considering 72 vs. 24 h (*p* < 0.0001 for 24 vs. 72 h). When comparing 48 vs. 72 h, DMCRT was more potent at 72 h for concentrations 1.43–5.71 mg/mL, whereas when considering the highest concentration of 11.43 mg/mL the effect was already maximized at 48 h of exposure (Figure 3A–G).

An interesting effect, concerning a “rescue” point of A172 cells, was observed, when cells were exposed at the lowest concentrations of DMCRT 0.18–0.36 mg/mL, as they gradually recovered at 72 h from the effects of DMCRT at 48 h (Figure 3F,G). The same effect was observed for cells exposed to CRCs at 0.71 mg/mL, as at 72 h, they slightly recovered from the compound’s cytotoxic effect (Figure 3E). However, although cells partially recovered at 72 h, attempting to overcome the cytotoxic effect of the tested compounds at 48 h, they do not completely succeed when compared with the effect at 24 h.

**Figure 4:**
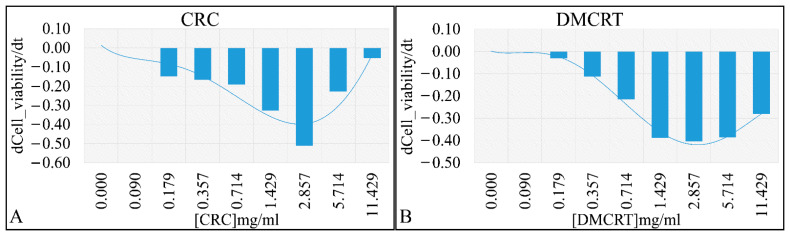
The “speed” by which CRCs and DMCRT reduce cell viability. CRCs manifested a dose-dependent effect from 0–2.857 mg/mL, indicating that the induced “damage” was time dependent. The 5.174 and 11.429 mg/mL concentrations showed that probably all effects were already induced at 24 h and time did not play a role thereafter (**A**). Similarly, DMCRT manifested a dose-dependent increase in “velocity” yet it reached a plateau after 2.857 mg/mL, indicating that its effects were mostly time-dependent in all cases (**B**) (**Legend**: **CRCs**: Crocins, **DMCRT**: dimethylocrocetin).

Based on the previous observations, we calculated the more efficient concentration of each phytochemical with respect to cell viability reduction velocity. Of note, the “speed” of alterations in cell viability is not identical to the effectiveness of a specific concentration, but demonstrates how fast a specific concentration could alter cellular viability observed at 24 h to the viability at 72 h. The concentration of 2.857 mg/mL for both compounds, manifested such effectiveness. In particular, CRCs demonstrated the local maximum at the 2.857 mg/mL concentration (Figure 4A). DMCRT manifested also a local maximum at the 2.857 mg/mL concentration, yet with comparable “speeds” for the 1.429 and 5.174 mg/mL concentrations (Figure 4B). Of note, negative values indicate that viability is lower at 72 h as compared to 24 h. A low first derivative, is compatible with higher effectiveness of the compound for a specific concentration. In addition, as the derivative approaches to zero, the more the compound exerted its effects from the first hours of treatment, whereas a derivative farther from zero, indicates that the compound requires more time in order to exert its effects. Thus, the effects of CRCs were changing dose-dependently for concentrations 0.09–2.86 mg/mL and thereafter CRCs’ action was already evident at 24 h of treatment (Figure 4A). On the contrary, DMCRT manifested a dose-dependent effect for concentrations 0.09–5.71 mg/mL, achieving a plateau thereafter, indicating that its effects remained time-dependent (Figure 4B). Interestingly, CRCs manifested one concentration with almost pure time-dependency (2.86 mg/mL), while DMCRT manifested three concentrations (1.43, 2.86, 5.71 mg/mL).

#### 3.2.4. The IC_50_ Curves of CRCs and DMCRT on Glioblastoma Cells (A172)

**Figure 5:**
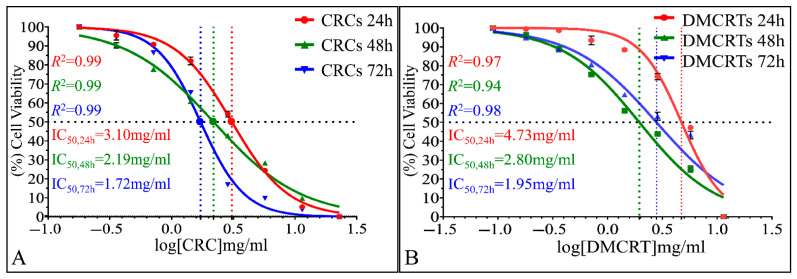
Calculation of IC_50_ of CRCs and DMCRT on A172 cells. IC_50_ were found 3.10 mg/mL (*R*^2^ = 0.99), 2.19 mg/mL (*R*^2^ = 0.99) and 1.72 mg/mL (*R*^2^ = 0.99), for CRCs, at 24, 48 and 72 h, respectively (**A**). IC_50_ for DMCRT were estimated 4.73 mg/mL (*R*^2^ = 0.97), 2.80 mg/mL (*R*^2^ = 0.94) and 1.95 mg/mL (*R*^2^ = 0.98) at 24, 48 and 72 h, respectively (**B**).

The time dependent effect of CRCs and DMCRT, as well as the superior in vitro sensitivity of A172 cells on CRCs were also depicted via IC_50_ calculations. IC_50_ were calculated 3.10 mg/mL (*R*^2^ = 0.99), 2.19 mg/mL (*R*^2^ = 0.99) and 1.72 mg/mL (*R*^2^ = 0.99), for CRCs, at 24, 48 and 72 h, respectively (Figure 5A). Further on, DMCRT manifested IC_50_ values of 4.73 mg/mL (*R*^2^ = 0.97), 2.80 mg/mL (*R*^2^ = 0.94) and 1.95 mg/mL (*R*^2^ = 0.98) at 24, 48 and 72 h, respectively (Figure 5B). 

The IC_50_ calculations confirmed our previous results and in particular, the fact that CRCs were more efficient, as compared to DMCRT. In addition, IC_50_ regressions produced better simulation results for CRCs, indicating that they exhibit superior time- and dose- dependent cytotoxicity, as compared to DMCRT.

#### 3.2.5. Gene Expression under CRCs and DMCRT on Glioblastoma Cells (A172)

**Figure 6:**
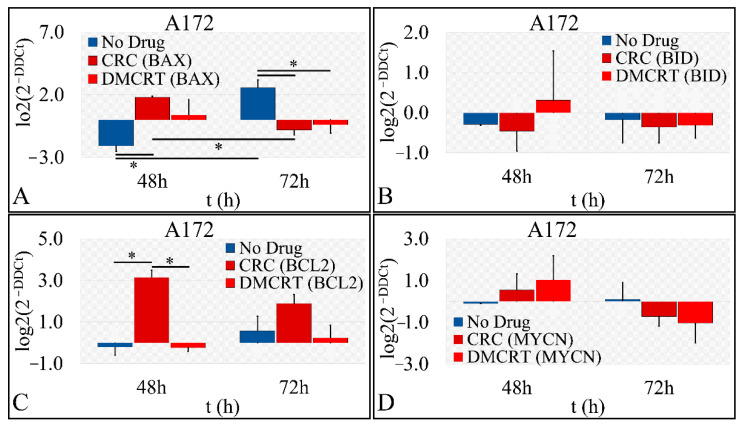

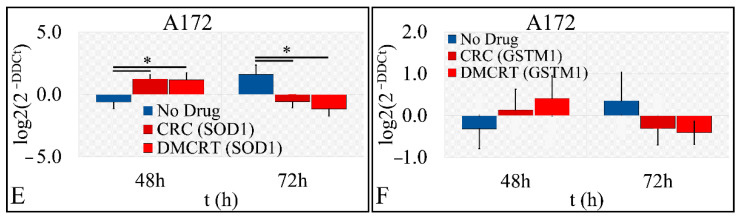
Gene expression under CRCs and DMCRT treatment. We performed indicative gene expression experiments for cells treated with CRCs and DMCRT. In particular, we investigated and compared to CRCs’ gene expression, gene expression for *BAX* (**A**), *BID* (**B**), *BCL2* (**C**), *MYCN* (**D**), *SOD1* (**E**) and *GSTM1* (**F**) (the asterisks depict a significance at *p* < 0.05 level).

Gene expression concerning the effects of CRCs and DMCRT on glioblastoma cells, was investigated and compared between the two compounds (Figure 6). Hence, gene expression was examined for *BAX* (Figure 6A), *BID* (Figure 6B), *BCL2* (Figure 6C), *MYCN* (Figure 6D), *SOD1* (Figure 6E) and *GSTM1* (Figure 6F) when cells were treated with 1 mg/mL CRCs and DMCRT. Although it is well established that exposure of cancer cells to concentrations of saffron or its compounds up to 16 mg/mL are related to apoptotic cell death and not necrosis, via alterations in the expression of relevant apoptotic genes, the dose treatment of 1 mg/mL was selected, based on the significant cytotoxic effects observed on both cell lines, without however complete cellular abolishment or exceeding the IC_50_ concentration for 48 and 72 h [62,63,64,65,66,67,68,69,70,71,72,73,74,75,76].

Initially, the putative apoptosis initiation mechanisms after treatment with saffron carotenoids were investigated, by measuring the expression of the *BAX*, *BID* and *BCL2* genes, which are involved in mitochondrial apoptotic pathways. *BAX* expression manifested significant differences between cells without treatment and cells exposed to CRCs or DMCRT at 48 and 72 h (Figure 6A). *BAX* was up-regulated at 48 h, whereas was down-regulated at 72 h, when cells were exposed to any of the compounds (Figure 6A). This effect signifies that saffron carotenoids exert a pro-apoptotic effect, from which cells attempt to recover later on. Similarly, the expression of the pro-apoptotic BID, which in response to apoptotic signaling interacts with BAX protein, facilitating its insertion into the mitochondrial membrane, was upregulated at 48 h of DMCRT treatment, whereas was down-regulated at 72 h for cells treated with CRCs or DMCRT, in accordance with *BAX* expression profile (Figure 6B). Further on, *BCL2* oncoprotein expression demonstrated significant differences between untreated cells and cells exposed to saffron phytochemicals. Although untreated glioblastoma cells do not strongly express the antiapoptotic BCL2 protein, *BCL2* was significantly up-regulated at 48 h when cells were treated with CRCs and at 72 h when cells were exposed to any of the carotenoids (Figure 6C). MYCN proto-oncogene was down-regulated at 72 h of treatment with any of saffron phytochemicals, although at 48 h it was upregulated (Figure 6D). Finally, another possible trigger of cellular apoptosis could be alterations in the expression of genes implicated in the antioxidant cellular machinery. Thus, *SOD1* (Figure 6E) and *GSTM1* (Figure 6F), both implicated in free radical scavenging, were down-regulated, at 72 h of treatment with CRCs or DMCRT, although initially upregulated at 48 h.

### 3.3. The Biological Effects on Rhabdomyosarcoma Cells (TE671)

#### 3.3.1. The Dose-Dependent Effect of CRCs and DMCRT on Rhabdomyosarcoma Cells (TE671)

**Figure 7:**
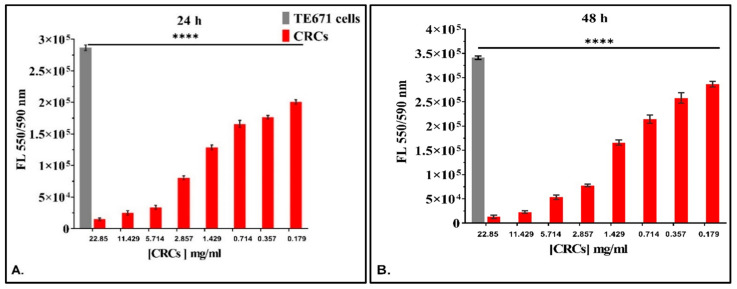

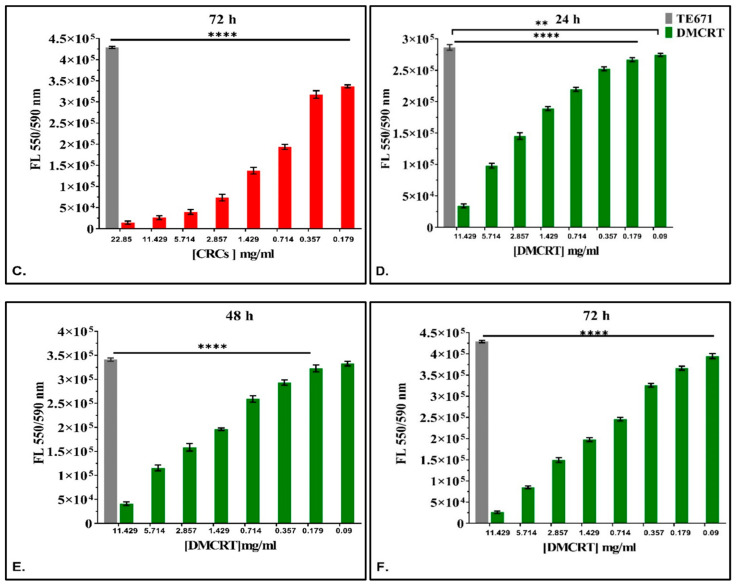
Dose-dependent effect of CRCs and DMCRT on TE671 cells. CRCs exerted a significant dose-dependent effect at all time intervals (*p* < 0.005 for cells exposed to any concentration of CRCs vs untreated TE671) (**A**–**C**). Similarly, DMCRT was also effective, manifesting significant increasing cytotoxicity, with increasing concentrations (**D**–**F**). **Legend**: **CRCs**: Crocins, **DMCRT**: dimethylocrocetin, **FL**: Fluorescence Intensity. Asterisks (*) depict at least *p* < 0.05 significance level between control experiments and CRCs (** *p* < 0.01; **** *p* < 0.0001). Each value represents the mean ± S.D. of triplicate experiments and CRCs (** *p* < 0.01; **** *p* < 0.0001). Each value represents the mean ± S.D. of triplicate experiments.

TE671 cells were incubated with various concentrations of CRCs and DMCRT. When analyzing drug effects with respect to concentration for each time interval studied (24, 48 and 72 h), CRCs and DMCRT were indifferent at 0 h of exposure, as expected (data not shown). Overall, both CRCs (Figure 7A–C) and DMCRT (Figure 7D–F) exhibited significant dose-dependent cytotoxicity at all-time points, when compared with untreated TE671 cells; as their concentration increased, cell viability was reduced with the exception of the lowest concentration of DMCRT (0.09 mg/mL) at 48 h.

#### 3.3.2. The Comparative Dose-Dependent Effect of CRCs and DMCRT on Rhabdomyosarcoma Cells (TE671)

**Figure 8:**
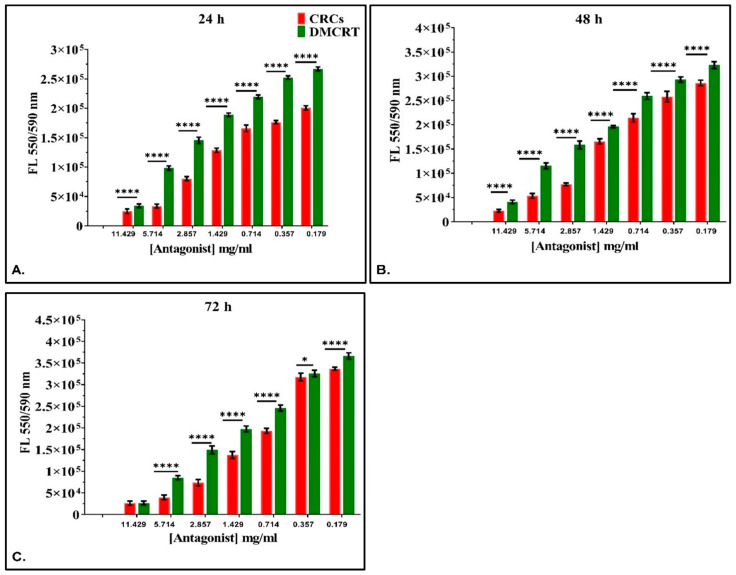
Comparative dose-dependent effect of CRCs and DMCRT on TE671 cells. The comparative effectiveness of CRCs and DMCRT, was investigated and presented together. CRCs and DMCRT followed a similar pattern at all time points and in particular CRCs exhibited higher efficacy than DMCRT. This effect was found to be true at all time points that is 24 h (**A**), 48 h (**B**) and 72 h (**C**) (**Legend**: **CRCs**: Crocins, **DMCRT**: dimethylocrocetin, **FL**: Fluorescence Intensity. Asterisks (*) depict at least *p* < 0.05 significance level between control experiments and CRCs (* *p* < 0.05; **** *p* < 0.0001). Each value represents the mean ± S.D. of triplicate experiments.

Similar to A172 cells, we examined the effect of saffron phytochemicals comparatively. Significant differences between CRCs and DMCRT were observed at all time points (24, 48 and 72 h) and for all concentrations, as CRCs manifested better cytotoxic effects, when compared to DMCRT (Figure 8). The exemption was the concentration of 11.43 mg/mL, at 72 h, in which their effect on TE671 did not differ.

#### 3.3.3. The Time-Dependent Effect of CRCs and DMCRT on Rhabdomyosarcoma Cells (TE671)

In addition to the previous analyses, the time-dependent efficacy of CRCs and DMCRT at each concentration tested was investigated. Measurements were initially performed at 6 h, meaning at the time just after the addition of the drug and the staining agent and every 24 h thereafter i.e., at 24, 48 and 72 h. At 6 h, as expected, no significant differences were present for any tested bioactive compound (data not shown).

**Figure 9:**
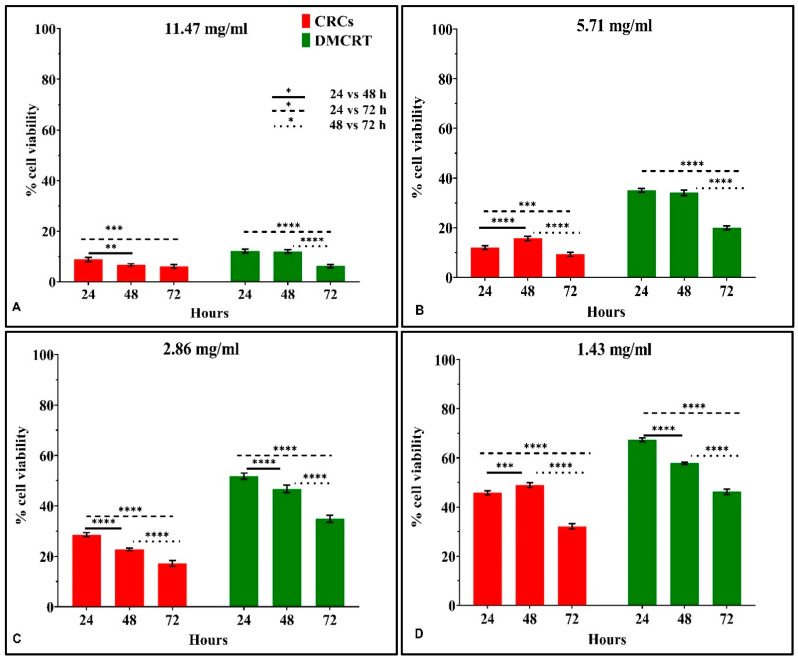

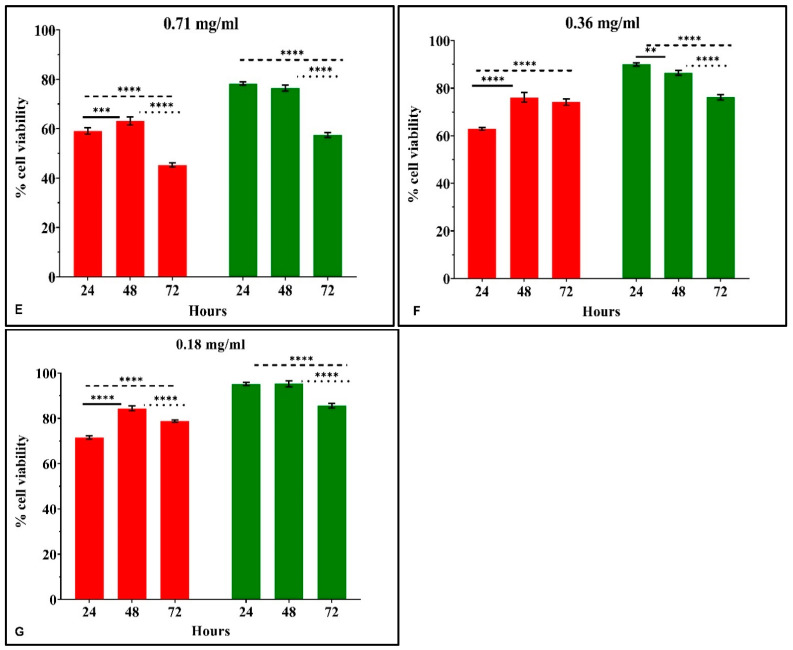
Time-dependent effect of CRCs and DMCRT on TE671 cells. Time-dependent effect of CRCs and DMCRT at concentrations 11.429 mg/mL (**A**), 5.174 mg/mL (**B**), 2.857 mg/mL (**C**), 1.429 mg/mL (**D**), 0.714 mg/mL (**E**), 0.357 mg/mL (**F**), 0.18 mg/mL (**G**) (**Legend**: **CRCs**: Crocins, **DMCRT**: dimethylocrocetin. Asterisks (*) depict at least *p* < 0.05 significance level between control experiments and CRCs (* *p* < 0.05; ** *p* < 0.01; *** *p* < 0.001; **** *p* < 0.0001). Each value represents the mean ± S.D. of triplicate experiments.

The time-dependent effect of CRCs on TE671 was more complicated as compared to A172 cells. CRCs at concentrations 0.71–11.43 mg/mL were more cytotoxic at 72 h, as compared to 24 h (*p* < 0.01 for 24 vs. 72 h). On the contrary, for the lowest concentrations of 0.18–0.36 mg/mL the effect of CRCs was more prominent at 24 h, when comparing 72 vs. 24 h, as cells partially recovered. When comparing 48 vs. 72 h, CRCs exhibited significant time-dependent cytotoxicity at 72 h for concentrations ≤ 5.71 mg/mL. The time dependent effect was not observed, when comparing 48 vs. 24 h, with the exception of concentrations 2.86 and 11.43 mg/mL as the cytotoxic effect of CRCs was more prominent at 24 h **(**Figure 9A–G).

DMCRT exhibited significant time-dependent cytotoxicity for all concentrations at 72 h, as compared to both 24 and 48 h (*p* < 0.01 for 72 vs. 24 or 48 h), manifesting a more stable cytotoxic effect with a gradual decline in cell viability. The same time-dependent effect was observed when comparing 24 and 48 h, for concentrations 1.43–2.86 mg/mL (*p* < 0.05 for 48 vs. 24 h), whereas for high concentrations ≥ 5.71 mg/mL the effect was already significant at 24 h (Figure 9A–G).

TE671 cells manifested a “clearer” “rescue” behavior at 48 h, when treated with CRCs at concentrations ≤ 1.43 mg/mL. Cell recovery at 48 h, indicated that cells attempted to overcome the cytotoxic effect of CRCs at 24 h, yet they do not succeed and cells continue to die, as demonstrated by the significant cytotoxicity at 72 h.

**Figure 10:**
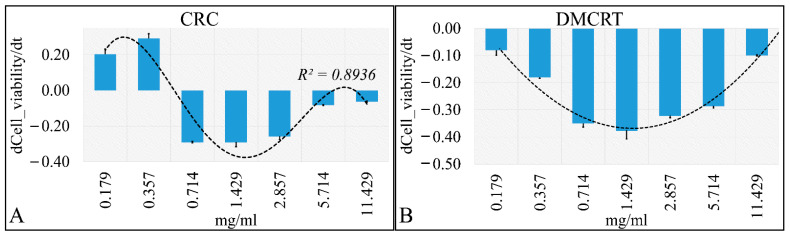
The “speed” by which CRCs and DMCRT reduces cell viability. CRCs manifested a threshold effect from 0.71–2.86 mg/mL, indicating that the induced cytotoxicity was time—independent (**A**). DMCRT manifested a dose-dependent “bell”-shaped behavior, with local maxima at 1.43 mg/mL, indicating that its effects were mostly time-dependent in all cases (**B**) (**Legend**: **CRCs**: Crocins, **DMCRT**: dimethylocrocetin).

Similar to A172 cells, the first derivative of cell viability with respect to individual concentrations was calculated. Cells exposed to CRCs manifested a higher cytotoxicity rate for concentrations between 0.714 mg/mL and 2.857 mg/mL (Figure 10A), whereas DMCRT manifested a quasi-normal distribution, with higher cell cytotoxicity rates at 1.429 mg/mL (Figure 10B). TE671 cells exposed to CRCs manifested three concentrations with similar effectiveness (0.71, 1.43 and 2.86 mg/mL), indicating three concentrations which are more time-dependent (Figure 9A). In addition, the cells manifested two concentrations (0.18, 0.36 mg/mL), with positive derivative, indicating that cells recovered during treatment (Figure 9A). At the same time, cells exposed to the higher concentrations (5.71, 11.43 mg/mL) manifested a derivative close to zero, indicating a dose-dependent effect, as cell viability at 72 h was close to cell viability observed at 24 h (Figure 9A). 

On the other hand, DMCRT manifested a normal-like distribution with respect to the first derivative (Figure 9B) and the most effective concentration identified was 1.43 mg/mL. Additionally, concentrations 0.71 and 2.86 mg/mL manifested similar behavior, indicating a time-dependent pattern of activity, whereas concentrations 0.18 and 11.43 mg/mL demonstrated a dose-dependent pattern (Figure 9B). DMCRT’s effect on TE671 cells, resembled the effect of DMCRT on A172 cells. The difference is that A172 cells manifested a slight shift of the normal-like curve towards the 2.86 mg/mL.

#### 3.3.4. The IC_50_ Curves of CRCs and DMCRT on Rhabdomyosarcoma Cells (TE671)

**Figure 11:**
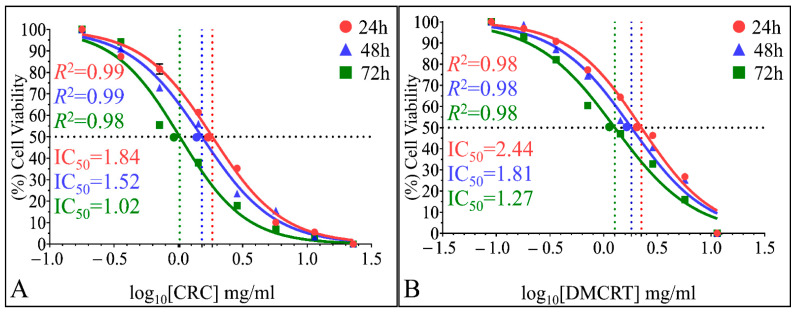
Calculation of IC_50_ of CRCs and DMCRT on TE671 cells. IC_50_ were found 1.84 mg/mL (*R*^2^ = 0.99), 1.52 mg/mL (*R*^2^ = 0.99) and 1.02 mg/mL (*R*^2^ = 0.98), for CRCs, at 24, 48 and 72 h, respectively (**A**). In addition, the IC_50_ for DMCRT was estimated to be 2.44 mg/mL (*R*^2^ = 0.98), 1.81 mg/mL (*R*^2^ = 0.98) and 1.27 mg/mL (*R*^2^ = 0.98) at 24, 48 and 72 h, respectively (**B**).

The time dependent effect of CRCs and DMCRT, as well the in vitro sensitivity of TE671 cells on CRCs were verified via IC_50_ calculations. IC_50_ were calculated 1.84 mg/mL (*R*^2^ = 0.99), 1.52 mg/mL (*R*^2^ = 0.99) and 1.02 mg/mL (*R*^2^ = 0.98), for CRCs, at 24, 48 and 72 h, respectively (Figure 11A). DMCRT manifested IC_50_ values of 2.44 mg/mL (*R*^2^ = 0.98), 1.81 mg/mL (*R*^2^ = 0.98) and 1.27 mg/mL (*R*^2^ = 0.98) at 24, 48 and 72 h, respectively (Figure 11B). In addition, IC_50_ regressions produced similar simulation results for CRCs and DMCRT.

#### 3.3.5. Gene Expression under CRCs and DMCRT on Rhabdomyosarcoma Cells (TE671)

**Figure 12:**
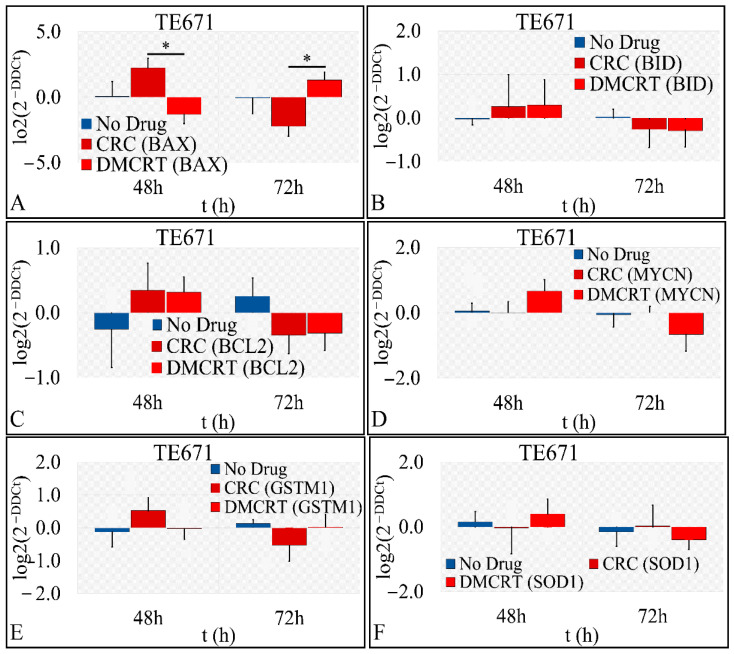
Gene expression under CRCs and DMCRT treatment for BAX (**A**), BID (**B**), BCL2 (**C**), MYCN (**D**), SOD1 (**E**) and GSTM1 (**F**) (* depicts a significance at the *p* < 0.05 level).

The expression of several genes, implicated in cellular apoptosis and survival and antioxidant defense mechanisms of TE671 cells, and treated with 1 mg/mL CRCs and DMCRT, was studied at a concentration which was close to the IC_50_ for both 48 and 72-h time intervals. Significant differences were observed in the case of BAX expression at 48 and 72 h of treatment. The apoptotic *BAX* was upregulated at 48 and 72 h for cells exposed to CRCs and DMCRT, respectively, whereas it was downregulated at 48 and 72 h for cells exposed to DMCRT and CRCs respectively (Figure 12A). On the other hand, *BID* was up-regulated at 48 h for cells treated with any of saffron phytochemicals but was down-regulated at 72 h (Figure 12B). The same expression pattern was observed when considering the pro-survival *BCL2* gene (Figure 12C). *MYCN* proto-oncogene was down-regulated at 72 h, only under DMCRT treatment (Figure 12D). When considering the antioxidant cellular mechanisms, CRCs downregulated *GSTM1* expression (Figure 12E), whereas DMCRT down-regulated *SOD1* (Figure 12F); both alterations were observed at 72 h. DMCRT and CRCs did not have any effect on *GSTM1* and *SOD1* expression, respectively, at any time point studied.

### 3.4. Functional Annotations of Selected Genes

**Figure 13:**
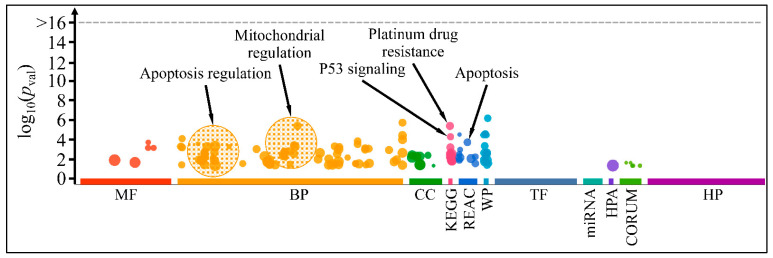
Gene ontology annotation of investigated genes (*BAX*, *BID*, *BCL2*, *MYCN*, *SOD1*, *GSTM1*). Major functions included apoptosis regulation, mitochondrial regulation, *p53* signaling, platinum drug resistance and apoptosis (**Legend**: **MF**: Molecular function; **BP**: Biological process; **CC**: Cellular component; **KEGG**: KEGG pathway database; **REAC**: Reactome pathway database; **WP**: WikiPathways; **TF**: Transcription factor binding motifs; **MIRNA**: miRNA targets; **HPA**: The human protein atlas; **CORUM**: The comprehensive resource of mammalian protein complexes; HP: Human phenotype ontology).

Functional annotation included gene ontology (GO) annotation of selected genes (Figure 13). This analysis preceded the gene expression analyses, as we have selected the investigated genes based on their interest and probable functions. Selected genes manifested major functions, such as apoptosis and mitochondrial regulation, whereas additional performances included cellular nitrogen utilization and response to toxic substances (Table S1). Interestingly, some of these genes, i.e., *BAX*, *SOD1* and *GSTM1*, manifested a dose- and time-dependent effect, in agreement with the observed cell viability and treatment.

## 4. Discussion

Medicinal plants, their secondary metabolites and synthesized chemical compounds have provided effective drug products for several diseases. The US National Cancer Institute has screened approximately 35,000 plant species for their potential anticancer action, founding that almost 3000 plant species have demonstrated significant and reproducible anticancer properties [77]. Among natural compounds, CRCs are endowed with antioxidant, anticancer and genoprotective properties, confirmed in in vitro and in vivo studies, such as on HL-60 human promyelocytic leukemia cells, K-562 human myelogenous leukemia cells, MCF-7 and MDA-MB-231 breast cancer cells, HO-8910 ovarian cancer cells, HeLa cervical carcinoma cells, A549 and SPC-A1 lung adenocarcinoma cells, BPH-1, LnCaP, 22rv1, CWR22, PC3, LapC-4 and PC3 prostate cells, MIAPaCa-2, BxPC3, Capan-1 and ASPC-1 pancreatic cancer cells, HepG2 hepatocellular carcinoma cells, AGS gastric cancer cells, HCT-116 SW-480 and HT-29 colorectal cancer cells, oral squamous cell carcinoma HN5, Ehrich sarcoma S-180, Erlich ascites carcinoma EAC and Dalton’s lymphoma DLA cells [74,75,78,79,80,81,82,83,84].

An important parameter that differentiates saffron carotenoids from current anticancer drugs is their selective cytotoxicity, as they inhibit cell proliferation of cancer cells, without affecting healthy cells, which is the cornerstone of effective cancer treatment [75,78,81,82,83,85,86,87,88]. Specifically, CRCs at doses of 9.77–48.85 μg/mL protected rat neural stem cells against hypoglycemia, and/or ischemia/reperfusion apoptosis via activation of the Notch signaling pathway, which induces neural stem cell proliferation and differentiation [89]. In dendritic cells CRCs at doses 1.25–5 mg/mL stimulated cell proliferation and promoted cell maturation [90]. Exposure of BV2 mouse microglial cells to 4.88–39.07 μg/mL of CRCs or 1.642–13.13 μg/mL of CRT protected cells from LPS (lipopolysaccharide)-induced cytotoxicity via downregulating the productions of intracellular reactive oxygen species (ROS), tumor necrosis factor-α and interleukin-1β [91]. Assessment of cell viability of neuro-2a mouse neuroblasts when exposed to CRCs for 24, 48 and 72 h at doses 24.424–195.392 μg/mL demonstrated significant positive effects on cell viability and attenuation of cellular apoptosis [92]. CRCs at doses of 0.078–0.156 mg/mL protected primary retinal cell cultures, containing a mixture of retinal cells enriched in photoreceptors, Müller and bipolar cells, against blue or white light–mediated apoptosis [93]. In normal human liver cell lines (L02 cells) CRT doses up to 13.13 μg/mL did not affect cell viability after 48 h of treatment and restored mitochondrial morphology and function in cells with FFA (free fatty acid)-induced lipid accumulation [94]. CRCs in a dose up to 0.977 mg/mL on human keratinocytes NHEKs (CC-2507) and dermal fibroblasts (C-013-5C) exhibited strong antioxidant capacity, comparable to vitamin E and higher than vitamin C, protecting against UVA-induced peroxidation and demonstrated significant anti-inflammatory properties [95]. Treatment of human normal fibroblastic cells (HFSF-PI3) with 1.9539–3.9078 mg/mL of CRCs or fibroblast mouse (L929) cells with 1–2 mg/mL of saffron ethanolic extract did not elicit any apoptogenic effect [11,96]. Similarly, CRCs in doses up to 0.088 mg/mL did not demonstrate any toxicity to the normal thyroid (PCCL3) cells [97]. CRCs in a dose of 1–4 mg/mL on breast epithelial MCF-10A cells did not affect the NF-κB-mediated inflammation and proliferation [98]. The cytoprotective properties of CRCs at doses up to 9.77 μg/mL were also demonstrated in bovine aortic endothelial cells (BAECs), exposed to significant oxidative stress [99,100]. CRCs at doses 9.77–39.079 μg/mL had no cytotoxic effect on primary bone marrow-derived macrophages (BMMs), even after 72 h of treatment [101]. T lymphocytes isolated from the peripheral blood of children with acute lymphoblastic leukemia (ALL) when treated with CRCs for 72 hours at a final concentration of 0.625–2.5 mg/mL exhibited a dose-dependent increase in their proliferation index and cytotoxic function [102]. Exposure of human umbilical vein endothelial cells (HUVEC) to saffron ethanolic or aqueous extracts and CRT at doses 0.1–1 mg/mL at various time intervals (24, 48 and 72 h) had no cytotoxic effects [103]. Saffron extracts at doses ranging from 50 to 400 μg/mL and 1 to 3 mg/mL had no effect on the viability of the human normal lung cells (CCD-18Lu) and non-cancer young adult mouse colon (YAMC) cells, respectively [84,104]. In normal fetal lung fibroblasts the IC_50_ of saffron extracts was calculated at 19.9 mg/mL, which was more than 10 times higher for tested human malignant cells, i.e., SKNSH neuroblastoma cells, HeLa cervical adenocarcinoma cells and MCF-7 breast cancer cells [105]. 

Retinoids, such as isotretinoin (13-*cis*-retinoic acid), ATRA (all-*trans* retinoic acid), etretinate and its metabolite acitretin, fenretinide (4-HPR), bexarotene and tazarotene, are used in the treatment of a broad spectrum of cancers, such as acute promyelocytic leukemia, ovarian carcinoma, head and neck squamous cell carcinoma, bladder cancer and neuroblastoma, due to their growth inhibitory, differentiating, antioxidant, anti-angiogenic, anti-inflammatory and immunomodulatory properties [11]. Nevertheless, significant toxicity and long-term storage in liver and adipose tissue may limit their usage at high therapeutic doses, outweighing their benefits in cancer treatment [106,107,108,109,110]. CRCs are fully water-soluble carotenoids, thus spreading easily throughout the body, reaching every tissue, whereas excess would be excreted via urine and not stored in tissues, such as adipose tissue or liver [20]. Thus, saffron’s carotenoids could represent an alternative to the toxicity of retinoids, harboring adequate anticancer, antioxidant and genoprotective properties [5,111,112].

### 4.1. In Vitro Cytotoxicity of Saffron’s Metabolites

In our study, in vitro results on glioblastoma A172 cells demonstrated that CRCs manifested a significant dose-dependent effect, at all-time points (24, 48 and 72 h); as their concentration increased, the reduction of resazurin of the staining agent to resorufin decreased, indicating reduction in cell viability. DMCRT’s cytotoxicity was dose dependent at 48 h and for concentrations ≥1.43 and ≥0.18 mg/mL, at 24 and 72 h, respectively. In the case of rhabdomyosarcoma TE671 cells CRCs exhibited significant dose-dependent cytotoxicity for all tested time points. DMCRT manifested a dose-dependent effect at 24- and 72 h and for concentrations ≥ 0.179 mg/mL at 48 h. For both cell lines, CRCs exhibited higher cytotoxicity, when compared with DMCRT.

CRCs on A172 cells manifested a time-dependent effect for all concentrations (11.43, 5.71, 2.86, 1.43, 0.71, 0.36 and 0.18 mg/mL) at 72 h, when compared with 24 h, whereas when compared with 48 h the time-dependent cytotoxicity was observed for concentrations ≥ 2.86 mg/mL. The absence of a time dependent effect between 72 vs. 48 h at the lowest concentrations 0.18–1.43 mg/mL could be attributed to the cytotoxicity effect reaching a plateau after 48 h of exposure. The time-dependent effect was also observed when comparing 48 and 24 h at concentrations ≤ 2.86 mg/mL, whereas for higher concentrations significant cytotoxicity was already present at 24 h. The time-dependent effect was clearer in the case of DMCRT; when comparing 48 vs. 24 h for all concentrations (11.43, 5.71, 2.86, 1.43, 0.71, 0.36 and 0.18 mg/mL) a significant reduction in cell viability was observed, as the time of exposure increased. The time-dependent effect was evident for concentrations ≥ 0.36 mg/mL, when considering 72 vs. 24 h, since the lowest concentration of 0.18 mg/mL did not produce any further cytotoxic effect, as it was demonstrated from dose-dependent analysis of DMCRT on A172 cells (Figure 1F). When comparing 72 vs. 48 h, the time-dependent result was evident at concentrations 1.43–5.71 mg/mL, whereas at lowest concentrations 0.18–0.36 mg/mL cells recovered at 72 h from the effects of DMCRT at 48 h, probably because the low concentrations could not sustain a prolonged cytotoxic effect.

For the TE671 cells the time-dependent efficiency was also evident for saffron’s carotenoids at most concentrations. CRCs demonstrated time-dependent cytotoxicity at 72 h, when compared with 24 h at concentrations 0.71–11.43 mg/mL, whereas the lowest concentrations 0.18–0.36 mg/mL could not sustain a prolonged cytotoxic effect. When comparing the activity of CRCs on TE671 cells at 72 vs. 48 h, the time-dependent effect was evident for concentrations ≤ 5.71, as the highest concentration of 11.43 mg/mL produced significant cellular alterations and cytotoxicity at 24 h, reaching a plateau at 48 h. In the case of DMCRT, a clear time -dependent cytotoxic action for all concentrations at 72 h was observed, when comparing both with 24 and 48 h, as it was evident with A172 cells. When comparing 48 vs. 24 h, a time-dependent cytotoxicity was observed, however was not significant, with the exclusion of concentrations near the calculated IC_50_ for 48 h (1.43–2.86 mg/mL), as the cytotoxic effect was maximized at 24 h, whereas sustained exposure for 72 h was needed to produce further significant cytotoxicity. 

The observed partial cellular recovery, i.e., for concentrations ≤1.43 mg/mL and ≤0.18 mg/mL of CRCs and DMCRT respectively at 72 vs. 48 h for A172 cells, ≤1.43 mg/mL and ≤0.36 mg/mL of CRCs, at 48 vs. 24 h and 72 vs. 48 h respectively for TE cells could be associated with a possible cytostatic effect of CRCs, causing cell cycle arrest at the G_0_/G_1_ phase, an effect already studied in several cancer cell lines [74,75,82,113,114,115]. Cells which are blocked in the G_0_/G_1_ phase successfully repair DNA lesions and are able to re-enter the cell cycle in a process called checkpoint recovery, allowing cell survival and proliferation. This phenomenon suggests a possible acquired resistance of cancer cells to saffron metabolites, as observed during usual chemotherapy and radiation therapy, attenuating their effect and leading to chemoresistance and tumor recurrence [116,117,118]. 

The calculation of IC_50s_ demonstrated the time dependent effect of CRCs and DMCRT, as well the superior in vitro activity of CRCs on both cell lines and the sensitivity of TE671 cells, when compared with A172 cells for both carotenoids. More specifically, the IC_50_ values for both compounds for A172 cells were almost 2× and 1.5× higher at 24 and 48/72 h respectively, in comparison with the respective values for TE671 cells. The calculated IC_50_ are in accordance with other studies on several cancer lines and within the reference range of safety for normal cells (Table 2) [11,90,93,95,96,103,105]. To our knowledge, it is the first time that the aforementioned cell lines, are exposed to CRCs and DMCRT and relevant IC_50_ are calculated.

A challenge arising from cytotoxicity experiments of drugs and phytochemicals, is the projection of the calculated IC_50s_ to optimized dosing and the prediction of in vivo efficacy and safety, so as to facilitate drug development for future clinical trials. However, in order to predict in vivo efficacy and project optimized dosing for clinical trials it requires physiologically-based pharmacokinetic/pharmacodynamic (PK/PD) mathematical models. Of note, drug efficacy is a highly complex process which depends on several factors, including the xenobiotic’s physicochemical properties, the route of administration, the characteristics of the formulation and characteristics of the recipient, affecting the absorption, distribution, metabolism and excretion, e.g., cytochrome P450 enzymes (CYP) activity, liver or kidney diseases and underlying physiological properties of the gastrointestinal tract.

The safety of saffron and its metabolites have been evaluated in clinical trials of both adult and pediatric patients, administered mainly *p.o.*, in different dosages or formulations (14–450 mg of saffron, 5–30 mg of CRCs, or 7.5–37.5 mg of CRT) [143,144,145]. Administration of *Crocus sativus* L. stigma tablets (200 and 400 mg/day, for 7 days) in healthy adult volunteers, was safe, without significantly affecting any hematological, biochemical and coagulation parameters [146,147,148]. Adult patients suffering from mild-to-moderate depression and anxiety, when receiving 30–100 mg of dried saffron stigma in 4, 6, 8 or 12-week trials, demonstrated mild significant side effects, which resolved spontaneously, without any intervention [149,150,151,152,153,154,155]. Similarly, capsules of SAE, and/or CRCs were well tolerated in doses of 15 mg twice daily for 12 weeks in patients suffering from COPD (chronic obstructive pulmonary disease), PCOS (polycystic ovary syndrome), depression or schizophrenia [154,156,157,158]. Patients with Alzheimer’s disease (AD) who received saffron extract (30 mg/day) capsules for 12 months benefited significantly, in terms of cognitive improvement and management of associated neuropsychiatric disturbances, without adverse events [159,160]. A similar safety profile was demonstrated in other clinical trials on patients with AD who received a capsule saffron 30 mg/day for 12, 16, or 22 weeks [161,162]. Administration of 14 mg of a standardized saffron extract in an 8-week trial to children aged 12–16 years old, exhibited beneficiary antidepressant and anxiolytic effects and was adequately tolerated, without any adverse effects [163]. Additionally, the administration of CRCs tablets (20 mg) in healthy volunteers for one month did not elicit any major adverse events or affect hematological, biochemical and hormonal parameters [148]. CRCs at a dose of 30 mg/day for a period of 8 weeks in patients with metabolic syndrome was associated with a favorable antioxidant effect [26,164]. CRCs at doses 5–15 mg for 3 months in diabetic patients, suffering from diabetic maculopathy, had a significant hypoglycemic effect, decreased HbA1c and ameliorated oxidative stress and inflammation [165]. 

Interestingly, the pharmacokinetic profile in humans of saffron and its metabolites have not been adequately studied. In a recent report, *p.o.* administration of saffron extract (56 and 84 mg) resulted in high bioaccessibility of CRCs isomers and CRT; 40.77 and 66.67, respectively, even after degradation during salivary, gastric and duodenal digestion. However, only CRT was detected in the plasma instead of CRCs, confirming that CRCs isomers are subjected to intestinal hydrolysis in the epithelial cells, forming deglycosylase trans-CRT, which is subsequently absorbed by passive diffusion from the intestinal epithelium, before reaching the bloodstream [166,167,168]. The maximum concentrations (Cmax) achieved were 0.26 μg/mL at 60 min and 0.39 μg/mL and 90 min, following 56 and 84 mg administration of the saffron extract, respectively, indicating a dose-dependent response kinetics [169,170]. The results were similar to other studies, as the Cmax of CRT was calculated 0.28 μg/mL and 0.35 μg/mL following a single *p.o.* dose of 22.5 mg of G. jasminoides CRT and 16 mg of saffron CRT, respectively. Oral and intravenous administration in rodents of SFE (60 mg/kg, equivalent with 16.7 mg/kg of CRCs), rich in all-trans-crocin (27.8% *w/w*) was associated with Cmax of CRT 2.77 μg/mL in serum [167]. CRT in most clinical and in vivo studies was not detected after 3–4 h of administration, suggesting a relatively short half-life for CRT (~0.85 ± 0.04 h), although longer time of detection (~24 h) has been documented [169,171,172]. CRT does not accumulate in the plasma, even after repeated oral doses of CRCs for six days [173].

Collectively, doses of saffron up to 1.5 gr/day are considered safe, whereas mild adverse effects are reported with doses of more than 5 gr/day. Adverse effects include nausea, vomiting, diarrhea, vertigo, hematuria, hematemesis or melena, uterus bleeding [12]. Doses more than 10 gr/day are associated with the induction of abortion, whereas there is a study in which frequent exposure to saffron particles, even without ingestion, could cause miscarriage [174]. Overdose (12–20 g/day) may be fatal [24]. The oral LD50 of saffron was estimated 20.7 g/kg, when administered as decoction, whereas the LD50 value of oral administration of SAE or ethanolic saffron extract was 3.6–5 g/kg [155]. Intraperitoneal treatment with saffron stigma extract or petal extract was associated with an LD50 value of 1.6 g/kg and 6 g/kg, respectively [26,160,175,176,177]. The LD50 of CRCs is estimated at >3 g/kg [26,33,34,35,36,37,38,39,40,41,42]. 

With reference to the previous data, saffron and its major constituents present a rather safe pharmacokinetic profile, even in healthy volunteers, as according to the toxicity classification, substances with a LD_50_ value within the range of 0.5–5 g/kg are considered practically low-toxic and substances with LD_50_ > 5 g/kg may be considered practically non-toxic [23,25,147,178,179,180,181]. In the current study, although a wide range of concentrations was used, i.e., 0.179–22.85 mg/mL for CRCs and 0.09–11.429 for DMCRT, such high concentrations were also employed in studies of saffron metabolites on hormone-insensitive prostate cancer cells (22rv1, C4–2B, DU145, PC3 cells), lung adenocarcinoma cells (A549, SPC-A1), HL-60 leukemia cells, HCT116 colorectal carcinoma and gastric adenocarcinoma (BGC-823, SGC-7901) cells. Thus, it can be safely suggested that the lower to middle, in vitro administered doses in the present preclinical study, could also be applied in an in vivo experimental setup and clinical studies and the calculated IC50s are in agreement with several other studies on different cancerous cell lines (Table 2).

### 4.2. Gene Expression Alterations in Cancer Cell Lines in the Presence of Saffron’s Metabolites

Despite accumulating evidence demonstrating that saffron and its carotenoids may be promising anti-cancer and chemopreventive agents, mechanisms of their actions are still largely ambiguous. Our team suggested that CRCs and DMCRT at the dose of 1 mg/mL significantly enhanced apoptotic phenomena on both A172 and TE671 cell lines, by altering the expression of pro-apoptotic, pro-survival and detoxifying genes. 

In A172 cells CRCs mediated apoptosis at 48 h via *BAX* upregulation. At 72 h however, CRCs triggered apoptosis, probably mainly via *MYCN* downregulation, as *BAX* and *BID* were downregulated. DMCRT induced apoptosis at 48 h via *BID* and *BAX* overexpression and *BCL-2* downregulation. At 72 h *MYCN* downregulation was the main determinant of apoptosis of cells exposed to DMCRT. The antiapoptotic *BCL-2* was significantly up-regulated at 48 h when cells were treated with CRCs and at 72 h when cells were exposed to CRCs or DMCRT, an effect which could also indicate a cellular attempt to counteract the BAX/BID apoptotic stimuli. The downregulation of the pro-apoptotic proteins BAX/BID at 72 h could explain the recovery effect from the compounds’ cytotoxic action.

In TE671 cells CRCs exerted an apoptotic effect at 48 h via *BAX and BID* upregulation, which was at some level counteracted by *BCL-2*, overexpression, leading to the observed cell recovery in cytotoxicity experiments. At 72 h however, CRCs triggered apoptosis, probably mainly via *BCL-2* downregulation, as *BAX* and *BID* expression was also suppressed. DMCRT induced apoptosis at 48 h mainly via *BID* overexpression, whereas at 72 h *BAX* upregulation, with concomitant *BCL-2* and *MYCN*, downregulation could justify the stronger cytotoxic effect of DMCRT at this time point. Although some gene expressions did not manifest significant differences, they still presented with an ascending or a descending pattern with respect to treatment, which should be taken into consideration.

Several phytochemicals exert their anti-cancer effects via inducing apoptosis in cancer cells. Apoptotic pathways are initiated via different entry sites, e.g., at the mitochondria (mitochondrial or intrinsic pathway) and the plasma membrane by death receptor signaling molecules (receptor or extrinsic pathway). Saffron and its active constituents stimulate apoptosis by activating and/or upregulating the expression of caspases, important in the initiation and execution of apoptosis, e.g., caspases-1, 3, 4, 5, 6, 7, 8 and-9, in a time-dependent manner. CRCs in several cancer cell lines up-regulate the expression of the proapoptotic proteins Bax, p53 and FasR receptor (FAS receptor)/APO-1 (apoptosis antigen 1). Bax and BID integrate apoptotic signals by disrupting the mitochondrial membrane potential, thus triggering the mitochondrial apoptotic pathway, as they facilitate permeabilization of the mitochondrial outer membrane and the release of apoptogenic factors to the cytosol, such as cytochrome *c* (CytC), Smac/Diablo, apoptosis-inducing factor (AIF) and endonuclease G (Endo-G). The tumor suppressor protein p53 induces both mitochondria-mediated apoptosis by activating other apoptotic proteins of BCL-2 family (Bax, Puma, Noxa) and the death receptor/extrinsic apoptosis pathway by promoting Fas/APO-1 death receptor expression [182,183]. Bcl-2 mediates antiapoptotic signals, preserving mitochondrial integrity, thus serving as a cellular mechanism to evade apoptosis and is associated with resistance to chemotherapeutic drugs. Conversely, CRCs down-regulate the expression of the anti-apoptotic Bcl-2, thus modulating the BAX/Bcl-2 ratio [74,81,82,83,96,135,138,184,185]. The balance between Bcl-2 and BAX expression dictates cellular behavior towards pro-survival or pro-apoptotic conditions [72].

Most studies agree on the fact that *Bcl-2* and *Bax* manifest opposite expression patterns under treatment with saffron or its metabolites, i.e., *Bcl-2* is down-regulated and *Bax* up-regulated (Table 3). To the best of our knowledge, this is the first report concerning the effects of CRCs and DMCRT on glioblastoma and rhabdomyosarcoma tumor cells. Yet in two known studies concerning neuronal cells (PC12 cells rat pheochromocytoma, retinal ganglion cells), exposure to saffron compounds elicited similar patterns of *BCL2* and *Bax* expression, as to our study, i.e., *Bcl-2* upregulation and *Bax* downregulation, protecting cells against oxidative stress-induced cell apoptosis [186,187]. Thus, as indicated in Table 3, saffron metabolites’ action on *Bax* and *Bcl-2* expression is tissue and cellular specific or associated with cellular exposure to intense oxidative stress. In our study, cellular apoptosis at the dose of 1 mg/mL, could be initiated and executed by additional pathways, as indicated in other studies, i.e., suppression of STAT3 activation and p73-dependent FAS-FADD (Fas-Associated Death Domain) induction, leading to BID activation, disruption of DNA–protein interactions (e.g., topoisomerase II), thus inhibiting nucleic acid synthesis, inactivation of the pro-survival cascade of PI3K/AKT, decreased expression of several MAPK (ERK1/2, p38), downregulation of JAK/STAT pathway, up-regulation of p21/CIP1 and p27/KIP1 which bind to CDK2, preventing its kinase activity causing cell cycle arrest [88,113,187,188,189,190,191].

*MYCN* belongs to the MYC family of proto-oncogenes and transcription factors, encoding a helix-loop-helix (bHLH) domain-based protein, implicated in the progression of the cell cycle through the G1/S transition. *MYCN* represents a critical oncogene, maintaining the tumorigenic state in several cancer types, implicated in cancer initiation, progression and invasion [141,191,192]. C-Myc downregulation at 72 h in the presence of CRCs and DMCRT for A172 cells and in the presence of DMCRT for TE671 cells could represent another mode of saffron cytotoxicity and induction of apoptosis in a MYCN-dependent manner. Similarly, CRCs were found to abrogate c-Myc expression in BXPC3 and capan-2 pancreatic adenocarcinoma cells and Y-79 and WERI-Rb-1 human retinoblastoma cells [141,191]. Thus, saffron metabolites could convey apoptotic signals via several pathways. Further investigation is necessary to elucidate the molecular mechanism by which CRCs and DMCRT are implicated in apoptosis of neuronal tumor cells.

A commonly accepted mechanism for the antitumor action of saffron is the inhibitory effect on free radical chain reactions, acting as high-efficiency reactive oxygen species (ROS) scavengers. Saffron and its metabolites in non-cancerous cells reduce oxidative stress overload by protecting against mitochondrial dysfunction and increasing the activity of antioxidant enzymes [superoxide dismutase (SOD), catalase, the enzymes of glutathione thioredoxin system, i.e., glutathione peroxidase (GPx), glutathione reductase (GR) and glutathione-S-transferase (GST)], quenching free radicals [e.g., hydroxyl (^•^OH), superoxide (O_2_^•−^), nitric oxide (NO^•^), nitrogen dioxide (^•^NO_2_), peroxyl (RO_2_^•^), alkoxyl (RO-) and lipid peroxyl (LOO^•^)] and decreasing lipid peroxidation of cell membranes [MDA, 4-hydroxynonenal (HNE), 4-oxo-2-nonenal (ONE) and 2-propenal acrolein] [16,27,40,67,74,83,87,193,194,195,196,197,198,199,200,201,202,203,204]. In A172 cells both *SOD1* and *GSTM1* were down-regulated, at 72 h of treatment with CRCs or DMCRT, although initially upregulated at 48 h, probably as a cellular adaptation to avoid CRCs and DMCRT cytotoxicity. In TE671 cells *GSTM1* and *SOD1* expression was abrogated in the presence of CRCs and DMCRT, respectively, at 72 h of exposure. Saffron’s phytochemicals at 72 h of exposure mitigated cell’s ability to maintain oxidative equilibrium and defend against oxidative stress, thus initiating cascades of apoptosis [205,206,207,208]. 

Overall, our results agree with three possible pathways via which CRCs and DMCRT exert their cytotoxicity on glioblastoma and rhabdomyosarcoma cell lines: (a) enhancement of *BAX* and *BID* -mediated cellular apoptosis; (b) suppression of *MYCN* and *BCL-2* cellular survival; and (c) suppression of the expression of *GSTM1* and *SOD1* oxidative species scavengers.

## 5. Conclusions

Our research endeavors for the first time to study the possible cytotoxic effects of CRCs and their semisynthetic derivative DMCRT on a glioblastoma and rhabdomyosarcoma cell lines and alterations in pathways implicated in cellular apoptosis and survival. Both CRCs and DMCRT manifested significant cytotoxic effects on A172 and TE671 cells, probably by altering the expression of genes implicated in apoptosis, i.e., *BAX* and *BID*, or cellular survival, i.e., *MYCN* and *BCL-2* and detoxifying mechanisms, i.e., *SOD1* and *GSTM1*. Saffron carotenoids and their semisynthetic derivates, as they are not the precursors of vitamin A, could be employed to minimize retinoid exposure and toxicity, even in high-dose and long-term application, especially in sensitive patient groups, such as children and pregnant women. A reasonable further approach would be further deciphering the mechanisms underlying saffron’s phytochemicals effects on cancer and normal cell lines and applying in vivo experiments to models of high-risk rhabdomyosarcoma and GBM, before employing relevant clinical trials, as CRCs are considered safe phytochemicals, already tested in humans mainly for psychiatric disorders and metabolic syndrome with tolerable side effects.

## Figures and Tables

**Table 1:** Gene name, Accession No., Primers.

Gene Symbol	Gene Name	Accession No	Primer F (5′-3′)	Primer R (5′-3′)
*ACTB*	actin, beta	NM_001101.3	CTGTCCACCTTCCAGCAGATGT	AGCATTTGCGGTGGACGAT
*GAPDH*	glyceraldehyde-3-phosphate dehydrogenase	NM_001256799.2	TTGCCCTCAACGACCACTTT	CACCCTGTTGCTGTAGCCAAA
*BAX*	BCL2 Associated X, Apoptosis Regulator	NM_001291428.1	GGTTGTCGCCCTTTTCTA	CGGAGGAAGTCCAATGTC
*BID*	BH3 interacting domain death agonist	NM_001196.3	TCCTTGCTCCGTGATGTCTTTC	AAGCTCCTCACGTAGGTGCGTA
*BCL2*	BCL2 apoptosis regulator	NM_000633.2	GATGTGATGCCTCTGCGAAG	CATGCTGATGTCTCTGGAATCT
*MYCN*	MYCN proto-oncogene, bHLH transcription factor	NM_001293228.1	CCCCTGGGTCTGCCCCGTTT	GCCGAAGTAGAAGTCATCTT
*SOD1*	superoxide dismutase 1	NM_000454.4	GGATGAAGAGAGGCATGTTGGA	TAGACACATCGGCCACACCAT
*GSTM1*	glutathione S-transferase mu 1	NM_000561.3	ACTTGATTGATGGGGCTCAC	TCTCCAAAATGTCCACACGA

**Table 2:** Antiproliferative effect of CRCs and DMCRT on several cell lines.

Cancer	Cell Line	IC50	Range of Concentrations	Ref(s)
Bladder cancer	5637	0.2 mg/mL	0.05–4 mg/mL of SAE	[23,119]
Breast cancer	MDA-MB-231	0.5 mg/mL of SAE	0.1–1 mg/mL of SAE	[25,62,66,80,81,98,105,120,121,122]
		>0.195 mg/mL of trans-CRC-4	9.7696 μg/mL–0.976 mg/mL of trans-CRC-4	
	BT-474 HER2+	3.5 mg/mL at 24 h of CRCs	1–5 mg/mL of CRCs	
	MCF-7	0.35–0.78 mg/mL of SAE	0.1–1 mg/mL of SAE	
		>0.195 mg/mL of trans-CRC-4	9.7696 μg/mL–0.976 mg/mL of trans-CRC-4	
		3.5 mg/mL at 48 h of treatment with CRCs	1.5–6 mg/mL of CRCs	
		0.05 mg/mL of CRCs	0.01–0.2 mg/mL of CRCs	
		3.5 mg/mL of CRCs	2–5 mg/mL of CRCs	
		>4 mg/mL of CRCs	1–4 mg/mL of CRCs	
	BT-549	~4 mg/mL of CRCs	1–4 mg/mL of CRCs	
	MDA-MB-468	3–4 mg/mL of CRCs	2–5 mg/mL of CRCs	
		3 mg/mL of CRCs at 24 h 2.5 mg/mL of CRCs at 48 h 1.5 mg/mL of CRCs at 72 h	1–5 mg/mL of CRCs	
Cervical cancer	HeLa	2.3 mg/mL of saffron ethanolic extract	1–5 mg/mL of saffron ethanolic extract	[63,76,105,123,124,125,126]
		1.92 mg/mL of saffron extracts	0.25–4 mg/mL of saffron extracts	
		3–3.5 mg/mL of CRCs	0.976–9.7696 mg/mL of CRCs	
		0.072248 mg/mL of CRT	0.328–1.31 mg/mL of CRT	
	SiHa	3.9078 mg/mL of CRCs	0.0587–15.613 mg/mL of CRCs	
	Sensitive human cervical cancer cell line OV2008 cells	3 mg/mL of CRCs at 24 h 1.5–2.7 mg/mL of CRCs at 48 h 1–1.5 mg/mL of CRCs at 72 h	1–5 mg/mL of CRCs	
	Resistant human cervical cancer cell line C13	7 mg/mL of CRCs at 24 h 5 mg/mL of CRCs at 48 h 2.5 mg/mL of CRCs at 72 h	1–4 mg/mL of CRCs	
Colorectal cancer	SW480	>1.0 mg/mL of Crocus sativus extract	0.25–3 mg/mL of Crocus sativus extract	[67,104,127,128]
		0.977 mg/mL of CRCs	0.029–0.977 mg/mL of CRCs	
	HCT116	1.0 mg/mL of Crocus sativus extract	0.25–3 mg/mL of Crocus sativus extract	
		1.94 mg/mL of CRCs	0.977–4 mg/mL of CRCs	
		>0.293 mg/mL of CRCs	0.029–0.977 mg/mL of CRCs	
		4.89–7.82 mg/mL of CRCs	0.488–14.65 mg/mL of CRCs	
		0.052 mg/mL of CRT	0.328–1.31 mg/mL CRT	
	HT-29 human colon adenocarcinoma cells	>1.0 mg/mL of Crocus sativus extract	0.25–3 mg/mL of Crocus sativus extract	
		>0.293 mg/mL of CRCs	0.029–0.977 mg/mL of CRCs	
		0.39 mg/mL of CRCs	4.88 μg/mL–1.9539 mg/mL of CRCs	
		>40 μg/mL of CRCs	10–40 μg/mL of CRCs	
	Caco-2	~40 μg/mL of CRCs	10–40 μg/mL of CRCs	
	DHD/K12-PROb rat colon adenocarcinoma cells	0.97696 mg/mL of CRCs	4.88 μg/mL–1.9539 mg/mL of CRCs	
Cutaneous squamous cell carcinoma	A431	3.9078 mg/mL of CRCs	0.977–3.9078 mg/mL of CRCs	[129]
	SCL-1	3.9078 mg/mL of CRCs	0.977–3.9078 mg/mL of CRCs	
Esophageal squamous carcinoma	KYSE-150	65.68 μg/mL of CRT	4.105–65.68 μg/mL of CRT	[130,131]
Gastric cancer	AGS	2.026–4 mg/mL of CRCs	0.003–19.68 mg/mL of CRCs	[48,69,70,132,133]
		2 mg/mL of CRCs	2–6 mg/mL of CRCs	
		2.405 mg/mL of CRCs	0.003–19.68 mg/mL of CRCs	
	SGC-7901	2.527 mg/mL of CRCs	0.003–19.68 mg/mL of CRCs	
	HGC-27	2–4 mg/mL of CRCs	2–6 mg/mL of CRCs	
	BGC-823	2.321 mg/mL of CRCs	8–16 mg/mL of CRCs	
	EPG85-257	~78.15 μg/mL	9.77–97.7 μg/mL	
Head and neck	HN5	0.58 mg/mL of CRCs at 48 h 0.48 mg/mL of CRCs at 72 h 12.5–50 µg/mL of CRCs for 24, 48 and 72 h had no inhibitory effects on cell proliferation	12.5–1000 μg/mL of CRCs	[45,132]
Hepatocellular carcinoma	HepG2	3 mg/mL of CRCs	0.977–5 mg/mL of CRCs	[64,71,125]
		2.75–3.25 mg/mL of CRCs at 48 h	0–10 mg/mL of CRCs	
		0.2 mg/mL CRT	0.328–1.31 mg/mL of CRT	
	HCCLM3	3 mg/mL of CRCs	3–5 mg/mL	
Leukemia	HL60	5 mg/mL of CRCs at 24 h	0.625–10 mg/mL of CRCs	[25,72,73,134,135,136,137]
		3 mg/mL of CRCs at 48 h	0.625–10 mg/mL of CRCs	
		>0.0328 mg/mL of CRT	0.0016–0.0328 mg/mL of CRT	
		11–39 mg/mL of CRCs	Non applicable	
		7–30 mg/mL of DMCRT	Non applicable	
	MOLT-4 human T-cell leukemia cell line	>0.488 mg/mL of CRCs	0.0488–0.488 mg/mL of CRCs	
	Jurkat cells	2.5 mg/mL of CRCs at 24 h 1.25 mg/mL at 48 h of CRCs	0.625–10 mg/mL of CRCs	
	CO 88BV59-1 EBV-transformed B-lymphocyte	0.17 mg/mL of CRCs at 24 h 0.109 mg/mL of CRCs at 48 h 0.0774 mg/mL of CRCs at 72 h	0.195 μg/mL–0.195 mg/mL of CRCs	[42]
Lung adenocarcinoma	A549	170–380 μg/mL of SAE	100–800 μg/mL of SAE	[11,74,125,138]
		1.5 mg/mL of saffron ethanolic extract at 24 h 0.565 mg/mL of saffron ethanolic extract at 48 h	0.5–2 mg/mL of saffron ethanolic extract	
		5.3537 mg/mL of CRCs	0.977–4 mg/mL of CRCs	
		4–5 mg/mL	1–16 mg/mL of CRCs	
		0.134 mg/mL CRT	0.328–1.31 mg/mL of CRT	
		4–5 mg/mL	1–16 mg/mL	
	SPC-A1	4–5 mg/mL	1–16 mg/mL	
Neuroblastoma	SKNSH	1.66 mg/mL of saffron extracts	0.25–4 mg/mL of saffron extracts	[105]
Ovarian cancer	SK-OV-3	3.3 mg/mL of CRCs	0.977–4 mg/mL of CRCs	[125,139]
		0.0623 mg/mL CRT	0.328–1.31 mg/mL of CRT	
	A2780	>0.0781 mg/mL of CRCs	0.00977–0.0977 mg/mL of CRCs	
Oral squamous cell carcinoma	KB	1.9246 mg/mL of CRCs	0.0488–3.9078 mg/mL of CRCs	[68]
Osteosarcoma	MG63	1.95–3.9 mg/mL of CRCs	0.488–3.9 mg/mL of CRCs	[65]
	OS732			
Pancreatic cancer	BxPC-3	>10 mg/mL of CRCs	1–10 mg/mL of CRCs	[140]
Prostate cancer	Hormone-sensitive LAPC-4, CWR22 and LnCaP cells	4.2-> 8 mg/mL of aqueous saffron extracts	0.1–8 mg/mL of aqueous and alcoholic saffron extracts	
	Hormone-insensitive 22rv1, C4–2B, DU145, PC3 cells	0.8–7.9 mg/mL of alcoholic saffron extracts	0.1–8 mg/mL of aqueous and alcoholic saffron extracts	[75]
		0.254–0.928 mg/mL of CRCs	0.0977–3.91 mg/mL of CRCs	
Retinoblastoma	Y-79	0.0195–0.07815 mg/mL of CRCs	0.00488–0.07815 mg/mL of CRCs	[141]
	WERI-Rb-1	0.0195–0.07815 mg/mL of CRCs	0.00488–0.07815 mg/mL of CRCs	
Tongue Squamous Cell Carcinoma	Tca8113	0.218 mg/mL of CRCs	0.098–0.781 mg/mL of CRCs	[142]

**Table 3:** Summary of the effects of CRCs and CRT on BCL2 and BAX gene expression.

Cell Line	BCL2	Effect	Ref.
Normal human liver cell L02	↑	Anti-apoptotic	[94]
Zebrafish NAFLD (Non-Alcoholic Fatty Liver Disease) model	↑	Anti-apoptotic	[94]
MC3T3-E1 osteoblasts	↑	Anti-apoptotic (after treatment with dexamethasone)	[209]
PC12 cells rat pheochromocytoma	↑	Anti-apoptotic (after treatment with acrylamide)	[186]
Retinal ganglion cells (RGCs)	↑	Anti-apoptotic (after retinal ischemia/reperfusion-IR injury)	[39]
Rat neural stem cells	↑	Anti-apoptotic (after glucose deprivation or IR injury)	[89]
Bovine aortic endothelial cells	↑	Anti-apoptotic (after cells are exposed to H_2_O_2_)	[100]
T24 cell of transitional cell carcinoma of bladder (TCCB)	↓	Pro-apoptotic	[114]
EBV-Transformed B-Lymphocytes	↓	Pro-apoptotic	[42]
BXPC3 and Capan-2 pancreatic adenocarcinoma	↓	Pro-apoptotic	[191]
KYSE-150 cells esophageal squamous cell carcinoma	↓	Pro-apoptotic	[190]
Acute promyelocytic leukemia cells HL60, NB4 and primary APL cells	↓	Pro-apoptotic	[210]
HL60 acute promyelocytic leukemia cells	↓	Pro-apoptotic	[72]
Hep3B and HepG2 hepatoblastoma	↓	Pro-apoptotic	[47]
Gastric adenocarcinoma (AGS) cells	↓	Pro-apoptotic	[96]
Human multiple myeloma cells	↓	Pro-apoptotic	[188]
MCF-7 breast cancer cells	↓	Pro-apoptotic	[184]
Human prostate cancer cells (LAPC-4, PC3)	↓	Pro-apoptotic	[75]
Lung adenocarcinoma A549 and SPC-A1	↓	Pro-apoptotic	[74]
Normal human liver cell L02	↓	Anti-apoptotic	[94]
Zebrafish	↓	Anti-apoptotic	[94]
MC3T3-E1 osteoblasts	↓	Anti-apoptotic	[209]
PC12 cells rat pheochromocytoma	↓	Anti-apoptotic (after treatment with acrylamide)	[186]
Retinal ganglion cells (RGCs)	↓	Anti-apoptotic (after retinal ischemia/reperfusion-IR injury)	[39]
Rat neural stem cells	↓	Anti-apoptotic (after glucose deprivation or IR injury)	[89]
Bovine aortic endothelial cells	↓	Anti-apoptotic (after cells are exposed to H_2_O_2_)	[100]
EBV-Transformed B-Lymphocytes	↑	Pro-apoptotic	[42]
KYSE-150 cells esophageal squamous cell carcinoma	↑	Pro-apoptotic	[190]
Acute promyelocytic leukemia cells HL60, NB4 and primary APL cells	↑	Pro-apoptotic	[210]
HL60 acute promyelocytic leukemia cells	↑	Pro-apoptotic	[72,210]
Hep3B and HepG2 hepatoblastoma	↑	Pro-apoptotic	[47]
Gastric adenocarcinoma (AGS) cells	↑	Pro-apoptotic	[96]
MCF-7 breast cancer cells	↑	Pro-apoptotic	[184]
Human prostate cancer cells (LAPC-4, PC3)	↑	Pro-apoptotic	[75]
T24 cell of transitional cell carcinoma of bladder (TCCB)	↑	Pro-apoptotic	[114]
HL-60 Human Leukemia Cells	↑	Pro-apoptotic	[72]
Lung adenocarcinoma A549 and SPC-A1	↑	Pro-apoptotic	[74]
Human multiple myeloma cells	↑	Pro-apoptotic	[188]
BXPC3 and Capan-2 pancreatic adenocarcinoma	↑	Pro-apoptotic	[191]

## Data Availability

All of the data is contained within the article and Supplementary Materials.

## Supplementary Materials

The following supporting information can be downloaded at: www.mdpi.com/article/10.3390/antiox11061074/s1, Table S1. The detailed description of GO enrichment as derived from the gprofiler web-tool.
