# Supplementary material for: An In Vitro Study of Saffron Carotenoids: The Effect of Crocin Extracts and Dimethylcrocetin on Cancer Cell Lines
Source: Antioxidants (Basel). 2022 May 28;11(6):1074. doi: 10.3390/antiox11061074 (PMC9220052; doi:10.3390/antiox11061074)
Supplement: Supplementary file 1 [file antioxidants-11-01074-s001.zip › antioxidants-1662481-supplementary.pdf]

antioxidants

MDPI

Article

An In Vitro Study of Saffron Carotenoids: The Effect of Crocin Extracts and Dimethylcrocetin on Cancer Cell Lines

Kyriaki Hatziagapiou [1,2,*,†], Olti Nikola [1,†], Sofia Marka [3], Eleni Koniari [4], Eleni Kakouri [5], Maria-Eleftheria Zografaki [6], Sophie S. Mavrikou [3], Charalabos Kanakis [5], Emmanouil Flemetakis [6], George P. Chrousos [4], Spyridon Kintzios [3], George I. Lambrou [1,*], Christina Kanaka-Gantenbein [1] and Petros A. Tarantilis [5]

1 Choremeio Research Laboratory, First Department of Pediatrics, National and Kapodistrian University of Athens, Thivon & Levadeias 8, 11527 Athens, Greece; khatziag@med.uoa.gr; onikola@med.uoa.gr (O.N.); ckanaka@med.uoa.gr (C.K.-G.)
2 Physiotherapy Department, Faculty of Health and Care Sciences, State University of West Attica, Agiou Spiridonos 28, Egaleo, 12243 Athens, Greece; khatziag@med.uoa.gr
3 Laboratory of Cell Technology, Department of Biotechnology, Agricultural University of Athens, EU-CONEXUS European University, 11855 Athens, Greece; smarka@aua.gr (S.M.); sophie_mav@aua.gr (S.S.M.); skin@aua.gr (S.K.)
4 University Research Institute of Maternal and Child Health & Precision Medicine, UNESCO Chair on Adolescent Health Care, National and Kapodistrian University of Athens, Thivon & Levadeias 8, 11527 Athens, Greece; hkoniari@med.uoa.gr (E.K.); chrousge@med.uoa.gr (G.P.C.)
5 Laboratory of Chemistry, Department of Food Science & Human Nutrition, School of Food Biotechnology and Development, Agricultural University of Athens, EU-CONEXUS European University, 11855 Athens, Greece; elenikakouri@aua.gr (E.K.); chkanakis@aua.gr (C.K.); ptara@aua.gr (P.A.T.)
6 Laboratory of Molecular Biology, Department of Biotechnology, Agricultural University of Athens, EU-CONEXUS European University, 11855 Athens, Greece; mzografaki@aua.gr (M.-E.Z.); mflem@aua.gr (E.F.)
* Correspondence: khatziag@med.uoa.gr (K.H.); glamprou@med.uoa.gr (G.I.L.)
† These authors contributed equally to this work and should be considered first authors.

SOURCE	TERM_NAME	ADJ. P_VALUE	INTERSECTIONS
GO:MF	BH3 domain binding	0.000200168	BAX, BCL2
GO:MF	BH domain binding	0.000733425	BAX, BCL2
GO:MF	death domain binding	0.000733425	BAX, BCL2
GO: BP	**positive regulation of intrinsic apoptotic signaling pathway**	1.89054E-06	BAX, BID, BCL2, SOD1
GO: BP	**regulation of mitochondrial membrane potential**	4.11443E-06	BAX, BID, BCL2, SOD1
GO: BP	**positive regulation of apoptotic signaling pathway**	3.8264E-05	BAX, BID, BCL2, SOD1
GO: BP	**protein insertion into mitochondrial membrane involved in apoptotic signaling pathway**	8.32542E-05	BAX, BID, BCL2
GO: BP	**regulation of intrinsic apoptotic signaling pathway**	0.000104947	BAX, BID, BCL2, SOD1
GO: BP	**positive regulation of mitochondrial outer membrane permeabilization involved in apoptotic signaling pathway**	0.000146303	BAX, BID, BCL2
GO: BP	**regulation of nitrogen utilization**	0.000279587	BAX, BCL2
GO: BP	**regulation of mitochondrial outer membrane permeabilization involved in apoptotic signaling pathway**	0.000310662	BAX, BID, BCL2
GO: BP	protein insertion into mitochondrial membrane	0.00037691	BAX, BID, BCL2
GO: BP	**positive regulation of cell death**	0.000471175	BAX, BID, BCL2, MYCN, SOD1
GO: BP	establishment of protein localization to mitochondrial membrane	0.000507114	BAX, BID, BCL2
GO: BP	ovarian follicle development	0.000536304	BAX, BCL2, SOD1
GO: BP	neuron apoptotic process	0.00053806	BAX, BID, BCL2, SOD1
GO: BP	**nitrogen utilization**	0.000559091	BAX, BCL2
GO: BP	**response to toxic substance**	0.000564259	BAX, BCL2, SOD1, GSTM1
GO: BP	mitochondrial outer membrane permeabilization	0.000597993	BAX, BID, BCL2
GO: BP	release of cytochrome c from mitochondria	0.000664228	BAX, BID, BCL2
GO: BP	positive regulation of mitochondrial membrane permeability involved in apoptotic process	0.000810977	BAX, BID, BCL2
GO: BP	mitochondrial outer membrane permeabilization involved in programmed cell death	0.000891809	BAX, BID, BCL2
GO: BP	positive regulation of mitochondrial membrane permeability	0.000977827	BAX, BID, BCL2
GO: BP	**intrinsic apoptotic signaling pathway**	0.001054384	BAX, BID, BCL2, SOD1
GO: BP	regulation of mitochondrial membrane permeability involved in apoptotic process	0.00106919	BAX, BID, BCL2
GO: BP	positive regulation of membrane permeability	0.001216602	BAX, BID, BCL2
GO: BP	retinal cell programmed cell death	0.001397316	BAX, BCL2
GO: BP	response to axon injury	0.001738502	BAX, BCL2, SOD1
GO: BP	regulation of mitochondrial membrane permeability	0.001871725	BAX, BID, BCL2
GO: BP	extrinsic apoptotic signaling pathway via death domain receptors	0.002011561	BAX, BID, BCL2
GO: BP	positive regulation of mitochondrion organization	0.00223406	BAX, BID, BCL2
GO: BP	regulation of apoptotic signaling pathway	0.002399642	BAX, BID, BCL2, SOD1
GO: BP	neuron death	0.002399642	BAX, BID, BCL2, SOD1
GO: BP	positive regulation of developmental pigmentation	0.002607555	BAX, BCL2
GO: BP	protein insertion into membrane	0.002998294	BAX, BID, BCL2
GO: BP	female gonad development	0.002998294	BAX, BCL2, SOD1
GO: BP	regulation of membrane permeability	0.003092591	BAX, BID, BCL2
GO: BP	development of primary female sexual characteristics	0.003489463	BAX, BCL2, SOD1
GO: BP	intrinsic apoptotic signaling pathway in response to DNA damage	0.003699981	BAX, BID, BCL2
GO:CC	mitochondrial outer membrane	0.003834007	BAX, BID, BCL2
GO:CC	pore complex	0.004261987	BAX, BCL2
GO:CC	organelle outer membrane	0.005407208	BAX, BID, BCL2
GO:CC	outer membrane	0.005548796	BAX, BID, BCL2
GO:CC	mitochondrial envelope	0.00694808	BAX, BID, BCL2, SOD1
KEGG	**Platinum drug resistance**	4.13249E-06	BAX, BID, BCL2, GSTM1
KEGG	**Apoptosis - multiple species**	5.34016E-05	BAX, BID, BCL2
KEGG	**p53 signaling pathway**	0.000661918	BAX, BID, BCL2
KEGG	Amyotrophic lateral sclerosis	0.002693269	BAX, BID, BCL2, SOD1
KEGG	Sphingolipid signaling pathway	0.002876347	BAX, BID, BCL2

**KEGG**	**Apoptosis**	0.004286524	BAX, BID, BCL2
**KEGG**	Measles	0.004574794	BAX, BID, BCL2
**KEGG**	Necroptosis	0.006827122	BAX, BID, BCL2
**KEGG**	Hepatitis B	0.007217276	BAX, BID, BCL2
**KEGG**	Pathways of neurodegeneration - multiple diseases	0.007744505	BAX, BID, BCL2, SOD1
**KEGG**	Tuberculosis	0.009076859	BAX, BID, BCL2
**KEGG**	Pathways in cancer	0.011794425	BAX, BID, BCL2, GSTM1
**KEGG**	Epstein-Barr virus infection	0.013086894	BAX, BID, BCL2
**KEGG**	Human immunodeficiency virus 1 infection	0.015573539	BAX, BID, BCL2
**KEGG**	Lipid and atherosclerosis	0.016466087	BAX, BID, BCL2
**REAC**	Activation, translocation and oligomerization of BAX	3.15444E-05	BAX, BID
**REAC**	Intrinsic Pathway for Apoptosis	0.000195569	BAX, BID, BCL2
**REAC**	BH3-only proteins associate with and inactivate anti-apoptotic BCL-2 members	0.001134101	BID, BCL2
**REAC**	Activation of BAD and translocation to mitochondria	0.003304058	BID, BCL2
**REAC**	**TP53 Regulates Transcription of Genes Involved in Cytochrome C Release**	0.00597314	BAX, BID
**REAC**	**Apoptosis**	0.007504833	BAX, BID, BCL2
**REAC**	**Programmed Cell Death**	0.009161674	BAX, BID, BCL2
**REAC**	Activation of BH3-only proteins	0.013649586	BID, BCL2
**REAC**	TP53 Regulates Transcription of Cell Death Genes	0.029605634	BAX, BID
**WP**	Amyotrophic lateral sclerosis (ALS)	6.65E-07	BAX, BID, BCL2, SOD1
**WP**	TP53 Network	2.59376E-05	BAX, BID, BCL2
**WP**	Nanomaterial induced apoptosis	2.59376E-05	BAX, BID, BCL2
**WP**	Regulation of Apoptosis by Parathyroid Hormone-related Protein	3.50177E-05	BAX, BID, BCL2
**WP**	Host-pathogen interaction of human corona viruses - apoptosis	3.50177E-05	BAX, BID, BCL2
**WP**	Photodynamic therapy-induced AP-1 survival signaling.	0.000469452	BAX, BID, BCL2
**WP**	**Apoptosis**	0.002282975	BAX, BID, BCL2
**WP**	Viral Acute Myocarditis	0.002363801	BAX, BID, BCL2
**WP**	**Apoptosis Modulation and Signaling**	0.002888716	BAX, BID, BCL2
**WP**	Small cell lung cancer	0.003380728	BAX, BID, BCL2
**WP**	Overview of nanoparticle effects	0.00734644	BAX, BCL2
**WP**	Unfolded protein response	0.012861208	BID, BCL2
**WP**	Integrated Breast Cancer Pathway	0.01304607	BAX, BID, BCL2
**WP**	Hepatitis B infection	0.01304607	BAX, BID, BCL2
**WP**	miRNA regulation of p53 pathway in prostate cancer	0.022571838	BAX, BID
**WP**	Photodynamic therapy-induced HIF-1 survival signaling	0.028431074	BAX, BID
**WP**	Integrated Cancer Pathway	0.042143043	BAX, BCL2
**HPA**	spleen; cells in white pulp[β‰¥Medium]	0.047615873	BAX, BID, BCL2, SOD1, GSTM1
**CORUM**	BAX homo-oligomer complex	0.024838735	BAX
**CORUM**	BAX homo-oligomer complex	0.024838735	BAX
**CORUM**	BCL2-CISD2 complex	0.049670619	BCL2
**CORUM**	**p53-BCL2 complex**	0.049670619	BCL2
**CORUM**	CALM1-FKBP38-BCL2 complex	0.049670619	BCL2

Table S1. The detailed description of GO enrichment as derived from the gprofiler web-tool.
